# Prediction and large-scale analysis of primary operons in plastids reveals unique genetic features in the evolution of chloroplasts

**DOI:** 10.1093/nar/gkz151

**Published:** 2019-03-04

**Authors:** Noam Shahar, Iddo Weiner, Lior Stotsky, Tamir Tuller, Iftach Yacoby

**Affiliations:** 1 School of Plant Sciences and Food Security, The George S. Wise Faculty of Life Sciences, Tel Aviv University, Ramat Aviv, Tel Aviv 69978, Israel; 2 Department of Biomedical Engineering, The Iby and Aladar Fleischman Faculty of Engineering, Tel Aviv University, Tel Aviv 6997801, Israel; 3 The Sagol School of Neuroscience, Tel Aviv University, Tel Aviv 6997801, Israel

## Abstract

While bacterial operons have been thoroughly studied, few analyses of chloroplast operons exist, limiting the ability to study fundamental elements of these structures and utilize them for synthetic biology. Here, we describe the creation of a plastome-specific operon database (link provided below) achieved by combining experimental tools and predictive modeling. Using a Reverse-Transcription-PCR based method and published data, we determined the transcription-state of 213 gene pairs from four plastomes of evolutionary distinct organisms. By analyzing sequence-based features computed for our dataset, we were able to highlight fundamental characteristics differentiating between operon pairs and non-operon pairs. These include an interesting tendency toward maintaining similar messenger RNA-folding profiles in operon gene pairs, a feature that failed to yield any informative separation in cyanobacteria, suggesting that it catches unique traits of operon gene expression, which have evolved post-endosymbiosis. Subsequently, we used this feature set to train a random-forest classifier for operon prediction. As our results demonstrate the ability of our predictor to obtain accurate (84%) and robust predictions on unlabeled datasets, we proceeded to building operon maps for 2018 sequenced plastids. Our database may now present new opportunities for promoting metabolic engineering and synthetic biology in chloroplasts.

## INTRODUCTION

Plastids are cellular organelles mainly found in a diverse group of photosynthetic organisms (1). They originate from an endosymbiotic event (∼1.5 × 10^9^ years ago) in which an ancient cyanobacterium was engulfed and retained by a eukaryotic cell, giving the latter the benefit of producing its own energy from sunlight (2). Since the debut of this co-evolutionary interaction, the majority of cyanobacterial genes were either lost or horizontally transferred to the host’ nuclear genome, while the plastid genome (plastome) mainly retained house-keeping and photosynthesis-related genes (3,4). As a result, the plastid has become highly dependent on imported nucleus-encoded proteins to conduct basic operations, making it a non-autonomous organelle (4). Nevertheless, the plastid conserved many of its ancestral characteristics and genomic features, such as the circular genome structure, 70S bacteria-like ribosomes, plastid-encoded bacterial-like RNA polymerase (PEP) and the organization of genes in bacterial-like operon transcription units (5–7).

Operons are DNA units comprised of several genes under the control of a single promoter that often share a common function (8,9). Inferring the operon map of a particular organism is an important step toward understanding its genetic regulatory networks, and could contribute to gene annotation as well (10). Several recent studies have attempted to predict bacterial operons by utilizing supervised machine-learning algorithms trained on experimental data (11–16). These computational methods typically rely on features such as intergenic distances between adjacent genes (16), conservation of gene order (17,18), functional classifications (19,20) and differential RNA levels (12,13). Thus, bacterial operons are relatively well-defined (21–24) and can be found in several online databases (15,25–28).

Unlike bacteria, plastid operons have no available databases and were only studied by few. These studies mainly focused on higher-plant model organisms; in *Hordeum vulgare* (barley) the entire operon map was revealed by differential RNA sequencing (29), in *Nicotiana tabacum* (tobacco) part of the polycistronic transcripts were revealed using northern-blot (30), in *Spinacia oleracea* (spinach) the rpoBC and the psbB operons were discovered using northern-blot (31,32), whereas the adenosine triphosphate (ATP) synthase operon was suggested by comparing its gene content and order to its homologous ATP synthase gene-cluster in *Escherichia coli* (33). In algae, part of the *Chlamydomonas reinhardtii* operons were studied; several operons were revealed using northern blot (34), whereas two recent reports identified 16 and 22 polycistronic units by searching for consistency in overlapping RNA-sequencing reads in the intergenic regions between adjacent genes (35,36). However, since no large-scale analysis of chloroplast operons exists, the ability to identify them, distinguish their characteristics, and use these data for synthetic biology purposes remains limited.

Plastid gene expression differs from that of model bacteria (e.g. *E. coli, Bacillus subtilis* etc.) in several features; chloroplast transcripts are often subject to RNA editing and splicing (37), the role of transcription termination is significantly reduced (38,39), many non-coding RNAs are frequently transcribed (29,36) and the plastome is suggested to be fully transcribed (38,39). Moreover, the expression of genes often relies on specific RNA-binding proteins (e.g. the pentatricopeptide repeat family) that bind *cis* elements upstream from the START codon, thus obstructing the activity of exoribonucleases and stimulating translation by suppressing stem-loops that hamper ribosome binding (40–42). Additionally, polycistrons are often regulated by multiple promoters and are massively processed (43,44), resulting in the formation of different transcript isoforms that derive from a single primary transcription unit (41,45,46). Thus, the plastid operon structure has evolved considerably compared to classical bacterial operons. These differences have most likely affected the composition and characteristics of chloroplast operons and gave rise to unique features.

Subsequently, the ability to transform synthetic genes into plastids has had a major impact on the field of plant biotechnology as it offers significant benefits compared to nuclear transformation. Among these advantages are homologous recombination based site specific integration (which is not available in many plant and algae nuclei) (47,48), the absence of gene-silencing (49), relatively high expression of heterologous genes (50–52) and prolonged transformation stability in most crops due to maternal inheritance (47). A consequent advantage that is particularly relevant for this study is the option to utilize the plastid’ natural ability to express polycistrons and design vectors with multiple genes under the control of a single promoter; thus minimizing plasmid sizes and allowing the introduction of several metabolic related transgenes in a single transformation (53–56).

Since both basic scientific questions and synthetic biology aspirations are hindered by the lack of large-scale information on plastid operons, in this work we describe the creation of a plastome-specific operon map database, using a combination of experimental tools and predictive modeling. The full database can be found at: https://www.energylabtau.com/cppod.

## MATERIALS AND METHODS

See Supplementary methods for additional information.

### Construction of labeled dataset

#### Empirical operon detection by RT-PCR

**Figure 1.**
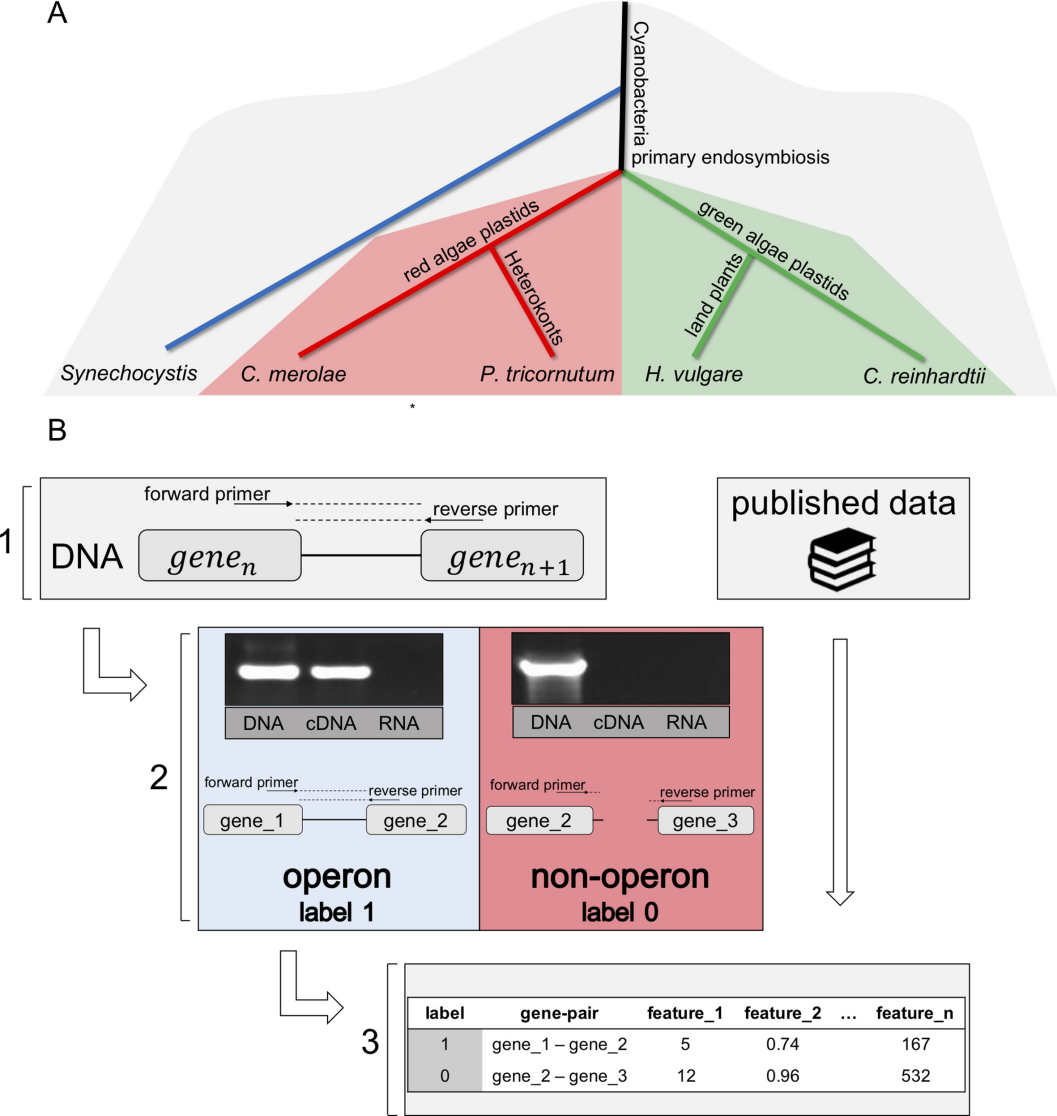
The creation of a generalist dataset of plastid operons comprised of distinct evolutionary organisms. (**A**) Phylogenetic tree of the plastomes and genomes used to train and test the model (based on (61)); (**B**) The main pipeline sketch; 1. Primer design for chosen gene pairs; 2. RT-PCR analysis—the DNA template is used to verify that the primers function properly, the RNA template is used to rule out DNA contamination in the cDNA samples and the cDNA reports on the transcription state of the pair (OP or NOP); 3. Sequence-based features are computed for each gene pair. Alternatively, known operon data are retrieved and their sequence-based features are directly computed.

**Figure 2.**
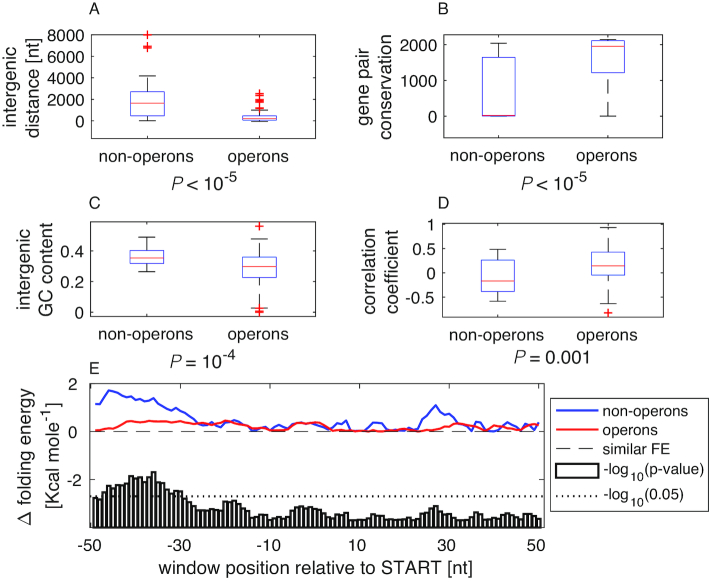
Operon sequence features. Distribution of (**A**) intergenic distances, (**B**) gene pair conservation, (**C**) intergenic GC content, (**D**) RNA structure similarity (Pearson’ correlation coefficient) among operon and non-operon gene pairs. (**E**) Position specific mRNA folding energy mean margin between gene pairs in absolute values. The bars represent the position specific *P*-value (-log_10_pv) comparing the OPs mean value to that of NOPs. The scale for the bar charts is not given, instead the common significance threshold is drawn (see legend). All *P*-values were computed using an unsupervised standard permutation test (see ‘Materials and Methods’ section: Permutation test). All significant *P*-values were confirmed using the Benjamini & Hochberg False Discovery Rate (FDR) procedure (62), ${N_{{\rm OP}}}$ = 137, ${N_{{\rm NOP}}}$ = 76.

To retrieve data on plastid operons for *Cyanidioschyzon merolae, Phaeodactylum tricornutum* and *C. reinhardtii*, each plastome (NC_004799, NC_008588 and NC_005353, respectively) was organized as a list of adjacent gene pairs. From each organism 20–40 gene pairs were selected for reverse-transcription PCR (RT-PCR) analysis. Specific primers were designed for each chosen gene pair; the forward annealed to the 5′ gene, whereas the reverse primer annealed to the 3′ gene (Figure 1B-1). DNA was extracted from cultures in standard growth conditions (see Supplementary Methods) using the chelex protocol (57). Total RNA was extracted using RNeasy plant Mini Kit (QIAGEN 74903). The purified RNA from each sample was used for complementary DNA (cDNA) synthesis using Applied Bio-System High-Capacity cDNA Reverse-Transcription Kit. Subsequently, for each of the aforementioned templates (i.e. DNA, RNA and cDNA) each gene pair was amplified by polymerase chain reaction (PCR) using its specific primers. The DNA template served as a positive control for the primers’ efficiency, the RNA template served as a negative control for denying the presence of plastid DNA and the cDNA template served as an indicator whether or not the gene pair is co-transcribed (Figure 1B-2). Only if the DNA control was positive and the RNA control was negative, the cDNA control was observed and labeled accordingly; one for the existence of a band, zero otherwise.

#### Collection of published operon data

The operon data of *H. vulgare* were taken from Zhelyazkova *et al.* (29, Supplementary Dataset S4). The operon data of *Synechocystis* sp. PCC6803 were taken from Kopf *et al.* (12, Supplementary Table S1). All other operon data of cyanobacteria (i.e. *Nostoc azollae, Acaryochloris marina, Cyanothece* sp. ATCC, *Trichodesmium erythraeum, Gloeobacter violaceus* PCC 7421 and *Synechococcus elongatus* PCC 6301) were retrieved from DOOR2 (15).

### RNA folding energy profiles

To create a RNA folding energy profile for each gene, we used a 40 nucleotide long sliding window and computed its free energy using the Vienna package (RNAfold) (58).

### Accuracy

\begin{equation*}{\rm accuracy }= \frac{{TP + TN}}{N}:\end{equation*}

Where: *TP* (True Positives) is the number of operons classified correctly, *TN* (True Negatives) is the number of non-operons classified correctly and *N* is the number of labeled observations in the dataset.

### Feature selection

A wrapped backward elimination feature selection was performed on the entire dataset. In this method our model’ mean accuracy over ten bootstrap samples was computed at the first part of each iteration of a random-forest classifier. In the second part, the feature/s with the lowest importance score was/were removed. Ultimately, a feature set that yielded high accuracy with a small set of features was manually selected (Supplementary Figure S5).

### Robustness test

**Figure 3.**
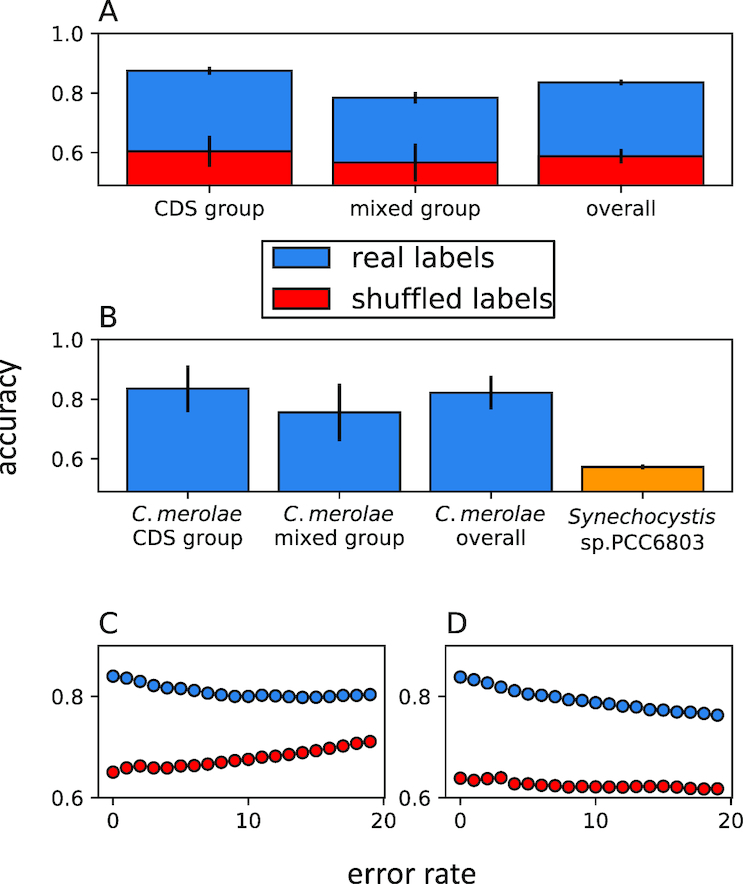
Model performance evaluation. (**A**) Accuracy scores of the random-forest classifier on real labels and random labels (see legend). ${N_{{\rm CDS}}}$ = 121, ${N_{{\rm mixed}}}$ = 92. (**B**) Prediction accuracies on the red alga *Cyanidioschyzon merolae* when discarded from the initial dataset, and on the cyanobacteria *Synechocystis* sp. PCC6803 labels derived from (12). The bars represent coding-sequence gene pairs (‘CDS group’), a mixture of tRNA, rRNA or coding sequence gene pairs (‘mixed group’) and the weighted mean of the two groups (‘overall’). *Synechocystis* sp. PCC6803 encompasses only CDS gene pairs. The bars show mean ± STD. (**C**) Type I (false-positive mistake) error test. (**D**) type II (false-negative mistake) error test. An overall of 19 errors were introduced into the labels and for each error rate the prediction pipeline was repeated. The same analysis was carried out on random labels. All accuracy scores were calculated based on the average accuracies of ten bootstrap trained samples.

The error test was carried out by randomly introducing 19 type I (false-positive mistake) or type II errors (false-negative mistake) into the labels and re-running the prediction pipeline with the selected features. For each error rate the mean accuracy score over ten bootstrap trained samples was calculated. For comparison, the same analysis was carried out on a permutated dataset as well (Figure 3C and D).

### Enrichment index

\begin{equation*}{\rm Enrichment}\,{\rm index}\ (i) = \frac{{\frac{{Xi}}{N} - \frac{{Ki}}{M}}}{{\frac{{Xi}}{N}}}\end{equation*}

Where *X* is the number of operon genes in the gene type *i, N* is the total number of operon genes, *K* is the total number (operon or not) of genes in the gene type *i* and *M* is the total number of genes in the dataset.

### Random-forest classifier

Initially, the dataset was divided into two groups: gene pairs that were comprised only of coding-sequences (CDS group) and a mixture of transfer RNA (tRNA), ribosomal RNA (rRNA) and CDS gene pairs (mixed group). Then each group was randomly split into a train set (70% of the data) and a cross-validation set (30% of the data) of gene pairs. A scikit-learn random-forest classifier (59,60) (‘n_estimators’ value was set to 1000 trees) was fitted based upon the train set and validated by predicting the cross-validation set. The process of training and cross-validating was repeated ten times, where in each round different cross-validation and train groups were selected. Each round of training and cross-validating yielded an accuracy score, representing the percentage of correct predictions on the cross-validation group (see ‘Materials and Methods’ section: Accuracy). The final accuracy score was the mean accuracy over ten bootstrap rounds of training and cross-validating.

### Large-scale plastid operon predictions

A total of 2018 plastomes were downloaded from the NCBI Organelle Genome Resources (see list of names and NCBI IDs in https://www.energylabtau.com/cppod) and features were calculated for each. Next, ten bootstrap classifiers (each classifier was trained and tested on different train and cross-validation groups) were used in order to predict labels for all organisms, where each gene pair was determined as ‘1’ or ‘0’ according to the majority voting of the classifiers. Finally, adjacent overlapping operon-pairs were concatenated to form full operon maps.

### Empiric *P*-value

The empiric *P*-value was computed as follows; fifty random samples were pooled from each group and their relevant traits were calculated. In each round the data were divided into two vectors (or groups) summarizing the outputs of all calculations performed on the extracted data, according to the hypothesis examined. If the average of the first vector was larger/smaller (depends on the objective function) than the average of the second vector, the current round received a ‘1’, otherwise ‘0’. Finally, the empiric *P*-value was calculated as the number of ‘1’ events divided by the number of total events.

### Permutation test

The *P*-values given in Figure 2 were computed using a permutation test, with no prior assumptions regarding the distribution of the data. In this test, all values were pooled and distributed *N* times into two random vectors, maintaining the original sizes of the original vectors. The margin between the means of the random vectors were compared to the original margin, where the fraction of margins more extreme than the original margin is the *P*-value. By default, *N* = 10^4^, but if this sample size yielded a fraction of 0, N grew to 10^5^. Due to computation time, if this sample size yielded a fraction of 0 as well, the *P*-value given was: *P* < 10^−5^.

## RESULTS

### Constructing an empirical dataset of primary transcription units

To create a generalist dataset of plastid operons, we began from obtaining empirical operon data from four chloroplast genomes: *H. vulgare* (higher plant), *C. reinhardtii* (green alga), *C. merolae* (red alga) and *P. tricornutum* (Heterokont) (Figure 1A) (61). For barley (*H. vulgare*), we used the published operon map that was revealed by RNA sequencing (29). For the other three species, we extracted total RNA from cultures in standard growth conditions and performed RT-PCR on a variety of gene pairs (see Supplementary Methods), where the forward primer annealed to the 5′ gene and the reverse primer annealed to the 3′ gene (Figure 1B-1). A DNA template was used to verify that the primers function properly, and the RNA template was used to rule out DNA contamination in the cDNA samples. Under these conditions, successful amplification of the cDNA reports that the two genes analyzed are present on a single RNA strand (i.e. Operon Pair, OP), whereas no amplification implies that the two are transcribed separately (i.e. Non-Operon Pair, NOP) (Figure 1B-2). Overall, we obtained operon data for 213 gene pairs (137 OPs and 76 NOPs) (Supplementary Table S1). To verify the reliability of the RT-PCR method, we performed the same test on the known psaA operon derived from the *H. vulgare* plastome (psaA-psaB-rps14-trnF-trnR, (29)) (Supplementary Figure S1A). The results clearly demonstrate that the RT-PCR is able to determine the actual transcription-state of a primary polycistron that is comprised of different types of genes (e.g. CDSs and tRNAs). Moreover, to prove that the RT-PCR method can robustly amplify cDNA transcripts with tight secondary structures (e.g. tRNAs), we successfully amplified the full cDNA transcript of six tRNA genes derived from the *C. reinhardtii* plastome (Supplementary Figure S1B).

For each gene pair we computed roughly 1100 features (Figure 1B-3 and Supplementary Figure S2) based on sequence analysis alone—thus, they could be computed for any sequenced plastome without requiring additional data (e.g. RNA-Seq). These features were designed to capture essential gene characteristics (e.g. coded protein hydrophobicity, RNA structure, codon usage bias, nucleotide composition) and to quantify their level of similarity within each couple of adjacent genes (Supplementary Figure S2).

Examination of these features yielded an array of indices, which created a significant separation between OPs and NOPs in our dataset (Supplementary Figure S3). As expected, intergenic distance and the gene pair conservation, which have been previously found to hold valuable information for operon prediction (15–18), could be used for meaningful classification in our dataset as well (Figure 2A and B). However, we were able to discover several novel informative features as well which are relevant to chloroplast operons but haven’t been reported before in the context of bacterial operons. One of these features shows that the GC content is significantly lower in intergenic spacers (IGSs) separating adjacent operon CDSs (i.e. transcribed spacers), compared to their non-operon counterparts (Figure 2C). Interestingly, we also found that neighbor operon CDSs are inclined to have similar RNA folding energy profiles in their 5′UTRs (Figure 2D and E). To validate the significance of these findings, we computed the *P*-value distribution of our entire feature set while shuffling its labels. We compared this distribution to that of the original data, and observed that the significance of the aforementioned features exceeds the limits of the randomized data (Supplementary Figure S4). Moreover, all significant P-values were confirmed using the Benjamini & Hochberg False Discovery Rate procedure (62).

To test whether these operon characteristics are shared with cyanobacterial operons, we computed the same features for all coding gene pairs derived from seven distinct cyanobacteria species. The transcription state of *Synechocystis* sp. PCC6803 was based on a comparative analysis of RNA-seq (12), whereas the operon data for the other six cyanobacteria were predicted by DOOR2 (15). Intergenic distance and gene pair conservation were found significant here as well, emphasizing the generalist nature of these operon traits (Supplementary Figure S3). Additionally, we observed that OPs tend to have low CDS or IGS GC content, both in chloroplasts and in cyanobacteria (Supplementary Figure S3). However, the messenger RNA (mRNA) folding profile similarity index failed to create any informative separation between OPs and NOPs (Supplementary Figure S3), indicating that this feature captures some of the unique traits for operon gene expression that have evolved in chloroplasts.

### Operon map inference

To create a model for chloroplast operon prediction, we used our labeled features to train a supervised random-forest classifier (59,60), where the objective was to predict whether or not a pair of adjacent genes are transcribed together. To increase the prediction accuracy and reduce the number of features used in the final model, we applied an iterative feature selection algorithm in which the least informative feature was removed at the end of each round (Supplementary Figure S5). Since some of the features were relevant to CDSs alone (e.g. codon usage bias), we ran this feature selection pipeline separately for CDS gene pairs and for all the rest (i.e. a mixture of CDS, tRNA and rRNA gene pairs). The final feature composition of our classifier can be seen in Supplementary Table S2.

To evaluate the performance of our model, we compared it to an array of random models in which the same data were used but the labels were permutated. Our overall accuracy, simply computed as the percentage of correct predictions for the cross-validation groups, was 84%—significantly higher (*P* < ${10^{ - 4}}$) than the random prediction (58%) (Figure 3A), with true positive and true negative rates of 87 and 79%, respectively (for the classifiers’ ROC curves and full metrics, see Supplementary Figure S6 and Supplementary Table S3). Pursuing a different approach, we discarded the red alga *C. merolae* from the initial dataset, re-performed the iterative feature selection, and applied this prediction to *C. merolae*. Our model showed roughly the same metrics in this case (Figure 3B and Supplementary Table S3), strengthening the relevance of its predictions on an organism that had not been a part of the learning process. To evaluate the uniqueness of our model to chloroplast operons, we used it to predict the operon map of *Synechocystis* sp. PCC6803 and compared it to its published operon map (12). The low accuracy score obtained (59%) highlights the orientation of our model toward predicting plastomic operons (Figure 3B).

To test the robustness of our prediction against false labels (which may occur due to rare PCR artifacts, sequencing errors etc.), we performed an error test by randomly introducing type I or type II errors into our labels and re-running the prediction pipeline. We observed that rates of up to 19 errors only reduced the accuracy score to around 80% (Figure 3C and D), thus ensuring that our model is sufficiently robust given a reasonable error rate in the original dataset. Finally, we applied our classifier to a large number of available sequenced chloroplast genomes (2018 plastomes).

### Plastid operon characteristics

**Figure 4.**
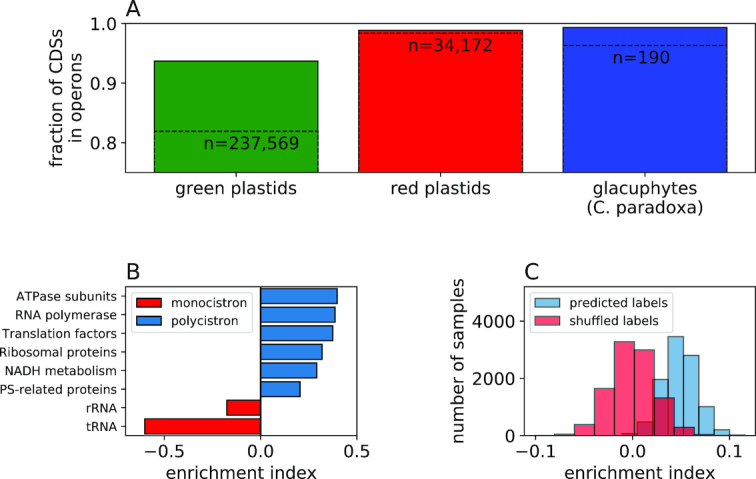
General traits of plastid primary transcripts. (**A**) The fraction of CDSs and total genes (dashed line) found in operons in green plastids, red plastids and glacuphytes. All differences return a non-significant empiric *P*-value of > $0.8$ (see ‘Materials and Methods’ section: Empiric *P*-value). The total number of genes sampled in each group is given above the relevant bar. The bars show mean ± SE. (**B**) Operon enrichment in different gene classes. Additionally, a hypergeometric enrichment/depletion *P*-value was computed for each group; *P* < 10^−300^ for all groups. (**C**) The distribution of functional enrichment in operons for 10 000 random samples shown in contrast to the same analysis carried on randomized data (see legend). Z-score and *P*-value (a two-tailed Wilcoxon test) between predicted labels and shuffled labels are 2.2 and $ < {10^{ - 300}}$, respectively.

By analyzing this newly formed database, we observed that indeed according to our predictor most chloroplast CDSs are transcribed as polycistrons (94.5 ± 0.05%), as suggested previously (29,47,63–65). We noticed that this ratio is roughly similar between green plastids (93.7 ± 0.06%), red plastids (98.86 ± 0.06%) and glacuphytes (*Cyanophora paradoxa*, 99.32%) (Figure 4A). To examine whether specific gene classes have different tendencies to be found in operons, we computed an enrichment index based on the hypergeometric distribution (methods: enrichment index). We observed that genes related to basic cell maintenance tend to be found in operons, while photosynthesis-related genes are found as monocistrons more frequently (Figure 4B). Interestingly, the RNA genes received the lowest enrichment scores, with tRNA having the strongest inclination toward the monocistronic form, as was hypothesized previously (5,29). To examine whether operons tend to be comprised of functionally related genes, we computed the same enrichment index on randomly-chosen samples of our database, and compared them to similar samples taken from a database with permutated labels. The results clearly show that primary operons in plastids tend to harbor functionally related genes (Figure 4C).

## DISCUSSION

### Model construction

Our choices in model selection were driven by our overall aspiration to construct a high-quality wide database of plastid primary operons. Thus, though other vascular plants—such as *N. tabacum*—have published operon data, we only used the barley operon map for two reasons: (i) it is highly reliable and encompasses all genes and (ii) we wished to avoid bias toward higher-plant plastid operons in our labeled data, as these are known to be highly conserved and redundant. In addition, we used the red alga *C. merolae* as an independent group to validate our model performances on a test set that had not been part of the initial dataset (Figure 3B and Supplementary Table S3). Furthermore, we chose to merge our empirical datasets and create a generalist plastid classifier (as opposed to using each representative for creating a group-specific classifier); using this wide perspective, our algorithm is able to grasp the main fundamental traits unique to operon genes, and avoid potential species-specific biases.

It is important to note that our empirical data were collected from cultures in normal growing conditions (see Supplementary Methods). Since it is known that operon maps might vary to some extent between different conditions (12,13), our database is likely to have the best predictive power for organisms in standard growing conditions. Subsequently, due to the nature of primary polycistronic processing in chloroplasts and the presence of multiple promoters, our database reflects the structure of primary operons (whether or not they undergo subsequent processing).

As described above, we decided to separate our predictor into a group where both adjacent genes are CDSs and another group that contains all the other combinations (i.e. CDS, tRNA and rRNA). We found the transcription state (NOPs or OPs) in this mixed group to be less predictable than that of CDSs (Figure 3A and B; Supplementary Table S3); one explanation for this is simply the loss of information induced by comparing two different types of genes (e.g. tRNA-CDS). Another phenomenon playing a role in explaining this observation is most likely the fact that tRNA and rRNA genes have less computable features (at least based on the approach employed here), compared to CDSs; while mRNAs tend to have structured and distinguishable sequence information dictated by the START to STOP flow, the codon triplet organization, and the constraints induced by the final protein sequence, extracting beneficial information from tRNA and rRNA genes is less trivial. For these reasons, existing works and tools neglect the tRNA and rRNA genes and target the CDSs alone when studying operons (12,13,15,25,27). In this work we included all genes, and although the CDS group received higher accuracy scores, the prediction accuracy was high in the mixed group as well—significantly outcompeting the random models (Figure 3A).

Since tRNAs are highly-structured and undergo 5′ cleavage for their proper maturation (66), we tested the credibility of our RT-PCR method in these cases. We were able to show that it properly detects the transcription state of mixed gene pairs (e.g. tRNA-CDS, Supplementary Figure S1A) and is able to amplify complete tRNA transcripts (Supplementary Figure S1B).

### Operon characteristics

Analysis of the features in our dataset highlighted interesting differences between chloroplast OPs and NOPs. Shorter intergenic spacer and lower transcript GC content in operon gene pairs (whether originating from the IGSs or from the CDSs themselves, Supplementary Figure S3) share the same logic; these are most likely means of sparing unnecessary resources. Since the IGS is fully transcribed, having a constraint on its length seems sensible in order to spare nucleotides, energy and transcription time. It also seems reasonable to have a constraint on the overall operon GC content in order to speed up transcription (67), as operon transcripts naturally tend to be long. Subsequently, minimizing the time-lag between the expression of adjacent operon genes is also beneficial since such pairs tend to serve similar functions (Figure 4C) and thus may often be required at roughly the same time. The presence of these features in both plastids and cyanobacteria (Supplementary Figure S3) highlights the generality of these concepts.

We were also able to identify a couple of operon features in plastids, which are not shared with cyanobacterial operons (Supplementary Figure S3). Interestingly, we found a high overall similarity in the mRNA folding profile at the 5′UTR of two adjacent operon genes (Figure 2D and E). The complete absence of this signal in cyanobacteria (Supplementary Figure S3) suggests that it captures unique traits that have evolved post-endosymbiosis. While mRNA folding is known to regulate gene expression on several levels (6,68–73), it is most likely that this characteristic is mainly related to translation, which unlike in bacteria—has become a major rate-limiting factor in chloroplast gene expression (42). Secondary mRNA structures affect both translation initiation, translation elongation and mRNA stability; thus, the similarity in folding energy profiles could be a powerful means for post-transcriptionally regulating operon genes toward simultaneous expression.

### Operons as a form of chloroplast translational regulation

After applying our classifier to a large number of sequenced plastomes, we organized the plastid genes into functional groups and examined the transcription state of the different groups. Our results show that the operon formation is widely preferred across all protein-coding groups, while RNA genes tend to be found in the monocistronic form (Figure 4B). This negligible role that operons play in regulation of plastid RNA gene expression could be explained by the overall high levels of transcription in chloroplasts (35,38,52) and the rate limiting nature of chloroplast translation. According to this hypothesis, co-transcription of functionally related CDSs is expected to be more beneficial as they are subject to similar translation regulation (Figure 2D and E). On the other hand, the expression of functionally related RNA genes as operons would not affect their expression synchronization, as they are not translated and are already highly expressed in the first place. Thus, assuming that operons were retained in plastids in order to co-regulate functionally related genes (Figure 4C), RNA genes (for which translation regulation is not relevant) could be expected to adopt the monocistronic form.

### CpPOD—an online database for predicted chloroplast operons

In this work, we have created a model that enables the prediction of primary plastid operons based on an annotated plastome alone. To make this ability useful, we ran our predictor on 2018 sequenced plastomes and uploaded the results to an open access site (see ‘Data Availability’ section). Besides binary final decision values (0 or 1), one can also find the predicted operon score, which is a continuous value between 0 and 1, reflecting the likelihood that the pair is co-transcribed. This allows the user to select stricter or more permissive thresholds than the default 0.5 threshold used in this work, if needed.

## CONCLUSION

In this work, we collected empirical operon data from plastomes of four model organisms, each representing a major plastid-containing group. Subsequently, we applied a supervised machine learning classification algorithm to build a generalist model for primary operon prediction that requires a sequenced plastome alone as input. By analyzing the features in our dataset, we were able to discover principle characteristics of operons, including their low overall GC content and similar mRNA folding patterns. Using a set of selected features, we were able to create a full database for predicted chloroplast operons that encompasses 2018 operon maps.

## DATA AVAILABILITY

The Chloroplasts Predicted Operon Database (CpPOD) is available online at: https://www.energylabtau.com/cppod.

## Supplementary Material

Supplementary Data

## References

[1] Sato N. Origin and evolution of plastids: genomic view on the unification and diversity of plastids. Struct. Funct. Plast. 2006; 23:75–102.

[2] Reyes-Prieto A., Weber A.P.M., Bhattacharya D. The origin and establishment of the plastid in algae and plants. Annu. Rev. Genet. 2007; 41:147–168. 17600460 10.1146/annurev.genet.41.110306.130134

[3] Barbrook A.C., Howe C.J., Purton S. Why are plastid genomes retained in non-photosynthetic organisms?. Trends Plant Sci. 2006; 11:101–108. 16406301 10.1016/j.tplants.2005.12.004

[4] Kleine T., Maier U.G., Leister D. DNA transfer from organelles to the nucleus: the idiosyncratic genetics of endosymbiosis. Annu. Rev. Plant Biol. 2009; 60:115–138. 19014347 10.1146/annurev.arplant.043008.092119

[5] Sugita M., Sugiura M. Regulation of gene expression in chloroplasts of higher plants. Plant Mol. Biol. 1996; 32:315–326. 8980485 10.1007/BF00039388

[6] Peled-zehavi H., Danon A. Translation and translational regulation in chloroplasts. Top. Curr. Genet. 2007; 19:249–281.

[7] Manuell A., Beligni M.V., Yamaguchi K., Mayfield S.P. Regulation of chloroplast translation: interactions of RNA elements, RNA-binding proteins and the plastid ribosome. Biochem. Soc. Trans. 2004; 32:601–605. 15270686 10.1042/BST0320601

[8] Fran B., Perrin D., Sanchez C., Monod J. The operon: a group of genes whose expression is coordinated by an operator < I loo loo. J. Bacteriol. 1960; 1729:1727–1729.

[9] Lawrence J. Selfish operons: the evolutionary impact of gene clustering in prokaryotes and eukaryotes. Curr. Opin. Genet. Dev. 1999; 9:642–648. 10607610 10.1016/s0959-437x(99)00025-8

[10] Brouwer R.W.W., Kuipers O.P., Van Hijum S.A.F.T. The relative value of operon predictions. Brief. Bioinform. 2008; 9:367–375. 18420711 10.1093/bib/bbn019

[11] Bockhorst J., Qiu Y., Glasner J., Liu M., Blattner F., Craven M. Predicting bacterial transcription units using sequence and expression data. Bioinformatics. 2003; 19:i34–i43. 12855435 10.1093/bioinformatics/btg1003

[12] Kopf M., Klähn S., Scholz I., Matthiessen J.K.F., Hess W.R., Voß B. Comparative analysis of the primary transcriptome of Synechocystis sp. PCC 6803. DNA Res. 2014; 21:527–539. 24935866 10.1093/dnares/dsu018 PMC4195498

[13] Fortino V., Smolander O.-P., Auvinen P., Tagliaferri R., Greco D. Transcriptome dynamics-based operon prediction in prokaryotes. BMC Bioinformatics. 2014; 15:145. 24884724 10.1186/1471-2105-15-145 PMC4235196

[14] Wolf Y.I., Rogozin I.B., Makarova K.S., Wolf Y.I., Koonin E. V Computational approaches for the analysis of gene neighbourhoods in prokaryotic genomes. Brief Bioinform. 2004; 5:131–149. 15260894 10.1093/bib/5.2.131

[15] Mao F., Dam P., Chou J., Olman V., Xu Y. DOOR: a database for prokaryotic operons. Nucleic Acids Res. 2009; 37:D459–D463. 18988623 10.1093/nar/gkn757 PMC2686520

[16] Salgado H., Moreno-Hagelsieb G., Smith T.F., Collado-Vides J. Operons in Escherichia coli: genomic analyses and predictions. Proc. Natl. Acad. Sci. U.S.A. 2000; 97:6652–6657. 10823905 10.1073/pnas.110147297 PMC18690

[17] Tamames J., Casari G., Ouzounis C., Valencia A. Conserved clusters of functionally related genes in two bacterial genomes. J. Mol. Evol. 1997; 44:66–73. 9010137 10.1007/pl00006122

[18] Overbeek R., Fonstein M., D’Souza M., Pusch G.D., Maltsev N. The use of gene clusters to infer functional coupling. Proc. Natl. Acad. Sci. U.S.A. 1999; 96:2896–2901. 10077608 10.1073/pnas.96.6.2896 PMC15866

[19] Riley M. Functions of the gene products of Escherichia coli. Microbiol. Rev. 1993; 57:862–952. 7508076 10.1128/mr.57.4.862-952.1993 PMC372942

[20] Romero P.R., Karp P.D. Using functional and organizational information to improve genome-wide computational prediction of transcription units on pathway-genome databases. Bioinformatics. 2004; 20:709–717. 14751985 10.1093/bioinformatics/btg471

[21] ten Broeke-Smits N.J.P., Pronk T.E., Jongerius I., Bruning O., Wittink F.R., Breit T.M., van Strijp J.A.G., Fluit A.C., Boel C.H.E. Operon structure of Staphylococcus aureus. Nucleic Acids Res. 2010; 38:3263–3274. 20150412 10.1093/nar/gkq058 PMC2879529

[22] Lim H.N., Lee Y., Hussein R. Fundamental relationship between operon organization and gene expression. Proc. Natl. Acad. Sci. U.S.A. 2011; 108:10626–10631. 21670266 10.1073/pnas.1105692108 PMC3127940

[23] Bratlie M.S., Johansen J., Drabløs F. Relationship between operon preference and functional properties of persistent genes in bacterial genomes. BMC Genomics. 2010; 11:71. 20109203 10.1186/1471-2164-11-71 PMC2837039

[24] Conway T., Creecy J.P., Maddox S.M., Grissom J.E., Conkle T.L., Shadid T.M., Teramoto J., Miguel P.S., Shimada T., Ishihama A. et al. Unprecedented high-resolution view of bacterial operon architecture revealed by RNA sequencing. MBio. 2014; 5:e01442-14. 25006232 10.1128/mBio.01442-14 PMC4161252

[25] Pertea M., Ayanbule K., Smedinghoff M., Salzberg S.L. OperonDB: a comprehensive database of predicted operons in microbial genomes. Nucleic Acids Res. 2009; 37:D479–D482. 18948284 10.1093/nar/gkn784 PMC2686487

[26] Klappenbach J.A. rrndb: the ribosomal RNA operon copy number database. Nucleic Acids Res. 2001; 29:181–184. 11125085 10.1093/nar/29.1.181 PMC29826

[27] Taboada B., Ciria R., Martinez-Guerrero C.E., Merino E. ProOpDB: prokaryotic operon database. Nucleic Acids Res. 2012; 40:D627–D631. 22096236 10.1093/nar/gkr1020 PMC3245079

[28] Salgado H. RegulonDB (version 4.0): transcriptional regulation, operon organization and growth conditions in Escherichia coli K-12. Nucleic Acids Res. 2004; 32:D303–D306. 14681419 10.1093/nar/gkh140 PMC308874

[29] Zhelyazkova P., Sharma C.M., Forstner K.U., Liere K., Vogel J., Borner T. The primary transcriptome of barley chloroplasts: numerous noncoding RNAs and the dominating role of the plastid-encoded RNA polymerase. Plant Cell. 2012; 24:123–136. 22267485 10.1105/tpc.111.089441 PMC3289561

[30] Shinozaki K., Ohme M., Tanaka M., Wakasugi T., Hayashida N., Matsubayashi T., Zaita N., Chunwongse J., Obokata J., Yamaguchi-Shinozaki K. et al. The complete nucleotide sequence of the tobacco chloroplast genome: its gene organization and expression. EMBO J. 1986; 5:2043–2049. 16453699 10.1002/j.1460-2075.1986.tb04464.x PMC1167080

[31] Hudson G.S., Holton T.A., Whitfeld P.R., Bottomley W. Spinach chloroplast rpoBC genes encode three subunits of the chloroplast RNA polymerase. J. Mol. Biol. 1988; 200:639–654. 3045324 10.1016/0022-2836(88)90477-9

[32] Westhoff P., Herrmann R.G. Complex RNA maturation in chloroplasts: the psbB operon from spinach. Eur. J. Biochem. 1988; 171:551–564. 2831053 10.1111/j.1432-1033.1988.tb13824.x

[33] Hennig J., Herrmann R.G. Chloroplast ATP synthase of spinach contains nine nonidentical subunit species, six of which are encoded by plastid chromosomes in two operons in a phylogenetically conserved arrangement. MGG Mol. Gen. Genet. 1986; 203:117–128.

[34] Rochaix J.D. Post-transcriptional regulation of chloroplast gene expression in Chlamydomonas reinhardtii. Plant Mol. Biol. 1996; 32:327–341. 8980486 10.1007/BF00039389

[35] Gallaher S.D., Fitz-Gibbon S.T., Strenkert D., Purvine S.O., Pellegrini M., Merchant S.S. High-throughput sequencing of the chloroplast and mitochondrion of Chlamydomonas reinhardtii to generate improved de novo assemblies, analyze expression patterns and transcript speciation, and evaluate diversity among laboratory strains and wild isolates. Plant J. 2018; 93:545–565. 29172250 10.1111/tpj.13788 PMC5775909

[36] Cavaiuolo M., Kuras R., Wollman F.A., Choquet Y., Vallon O. Small RNA profiling in chlamydomonas: Insights into chloroplast RNA metabolism. Nucleic Acids Res. 2017; 45:10783–10799. 28985404 10.1093/nar/gkx668 PMC5737564

[37] Germain A., Hotto A.M., Barkan A., Stern D.B. RNA processing and decay in plastids. Wiley Interdiscip. Rev. RNA. 2013; 4:295–316. 23536311 10.1002/wrna.1161

[38] Shi C., Wang S., Xia E.H., Jiang J.J., Zeng F.C., Gao L.Z. Full transcription of the chloroplast genome in photosynthetic eukaryotes. Sci. Rep. 2016; 6:1–10. 27456469 10.1038/srep30135 PMC4960489

[39] Legen J., Kemp S., Krause K., Profanter B., Herrmann R.G., Maier R.M. Comparative analysis of plastid transcription profiles of entire plastid chromosomes from tobacco attributed to wild-type and PEP-deficient transcription machineries. Plant J. 2002; 31:171–188. 12121447 10.1046/j.1365-313x.2002.01349.x

[40] Miranda R.G., Rojas M., Montgomery M.P., Gribbin K.P., Barkan A. RNA-binding specificity landscape of the pentatricopeptide repeat protein PPR10. RNA. 2017; 23:586–599. 28108520 10.1261/rna.059568.116 PMC5340921

[41] Pfalz J., Bayraktar O.A., Prikryl J., Barkan A. Site-specific binding of a PPR protein defines and stabilizes 5′ and 3′ mRNA termini in chloroplasts. EMBO J. 2009; 28:2042–2052. 19424177 10.1038/emboj.2009.121 PMC2718276

[42] Zoschke R., Bock R. Chloroplast translation: structural and functional organization, operational control and regulation. Plant Cell. 2018; 30:745–770. 29610211 10.1105/tpc.18.00016 PMC5969280

[43] Delannoy E., Stanley W.A., Bond C.S., Small I.D. Pentatricopeptide repeat (PPR) proteins as sequence-specificity factors in post-transcriptional processes in organelles. Biochem. Soc. Trans. 2007; 35:1643–1647. 18031283 10.1042/BST0351643

[44] Monde R., Schuster G., Stern D. Processing and degradation of chloroplast mRNA. Biochimie. 2000; 82:573–582. 10946108 10.1016/s0300-9084(00)00606-4

[45] Drechsel O., Bock R. Selection of Shine-Dalgarno sequences in plastids. Nucleic Acids Res. 2011; 39:1427–1438. 20965967 10.1093/nar/gkq978 PMC3045613

[46] Hammani K., Takenaka M., Miranda R., Barkan A. A PPR protein in the PLS subfamily stabilizes the 5′-end of processed rpl16 mRNAs in maize chloroplasts. Nucleic Acids Res. 2016; 44:4278–4288. 27095196 10.1093/nar/gkw270 PMC4872118

[47] Fuentes P., Armarego-Marriott T., Bock R. Plastid transformation and its application in metabolic engineering. Curr. Opin. Biotechnol. 2018; 49:10–15. 28738208 10.1016/j.copbio.2017.07.004

[48] Weiner I., Atar S., Schweitzer S., Eilenberg H., Feldman Y., Avitan M., Blau M., Danon A., Tuller T., Yacoby I. Enhancing heterologous expression in Chlamydomonas reinhardtii by transcript sequence optimization. Plant J. 2018; 94:22–31. 29383789 10.1111/tpj.13836

[49] Bock R. Engineering plastid genomes: methods, tools, and applications in basic research and biotechnology. Annu. Rev. Plant Biol. 2015; 66:211–241. 25494465 10.1146/annurev-arplant-050213-040212

[50] Jarvis P., López-Juez E. Biogenesis and homeostasis of chloroplasts and other plastids. Nat. Rev. Mol. Cell Biol. 2013; 14:787–802. 24263360 10.1038/nrm3702

[51] Jones C.S., Luong T., Hannon M., Tran M., Gregory J.A., Shen Z., Briggs S.P., Mayfield S.P. Heterologous expression of the C-terminal antigenic domain of the malaria vaccine candidate Pfs48/45 in the green algae Chlamydomonas reinhardtii. Appl. Microbiol. Biotechnol. 2013; 97:1987–1995. 22592550 10.1007/s00253-012-4071-7

[52] Weiner I., Shahar N., Feldman Y., Landman S., Milrad Y., Ben-Zvi O., Avitan M., Dafni E., Schweitzer S., Eilenberg H. et al. Overcoming the expression barrier of the ferredoxin-hydrogenase chimera in Chlamydomonas reinhardtii supports a linear increment in photosynthetic hydrogen output. Algal Res. 2018; 33:310–315.

[53] Zhou F., Karcher D., Bock R. Identification of a plastid intercistronic expression element (IEE) facilitating the expression of stable translatable monocistronic mRNAs from operons. Plant J. 2007; 52:961–972. 17825052 10.1111/j.1365-313X.2007.03261.x PMC2230500

[54] Lu Y., Rijzaani H., Karcher D., Ruf S., Bock R. Efficient metabolic pathway engineering in transgenic tobacco and tomato plastids with synthetic multigene operons. Proc. Natl. Acad. Sci. U.S.A. 2013; 110:E623–E632. 23382222 10.1073/pnas.1216898110 PMC3581966

[55] Legen J., Ruf S., Kroop X., Wang G., Barkan A., Bock R., Schmitz-Linneweber C. Stabilization and translation of synthetic operon-derived mRNAs in chloroplasts by sequences representing PPR protein-binding sites. Plant J. 2018; 94:8–21. 29418028 10.1111/tpj.13863

[56] Macedo K.S., Víctor O., España H.P., Garibay C., Daniel O., Zapata G., Durán N. V, Jesús F., Corona A.B. Intercistronic expression elements (IEE) from the chloroplast of Chlamydomonas reinhardtii can be used for the expression of foreign genes in synthetic operons. Plant Mol. Biol. 2018; 98:303–317. 30225747 10.1007/s11103-018-0776-z

[57] Cao M., Fu Y., Guo Y., Pan J. Chlamydomonas (Chlorophyceae) colony PCR. Protoplasma. 2009; 235:107–110. 19242652 10.1007/s00709-009-0036-9

[58] Lorenz R., Bernhart S.H., Höner zu Siederdissen C., Tafer H., Flamm C., Stadler P.F., Hofacker I.L. ViennaRNA Package 2.0. Algorithms Mol. Biol. 2011; 6:26. 22115189 10.1186/1748-7188-6-26 PMC3319429

[59] Pedregosa F., Varoquaux G., Gramfort A., Michel V., Thirion B., Grisel O., Blondel M., Prettenhofer P., Weiss R., Dubourg V. et al. Scikit-learn: machine learning in python. J. Mach. Learn. Res. 2012; 12:2825–2830.

[60] Liaw A., Wiener M. Classification and regression by randomForest. R News. 2014; 2:18–22.

[61] Keeling P.J. The endosymbiotic origin, diversification and fate of plastids. Philos. Trans. R. Soc. B Biol. Sci. 2010; 365:729–748. 10.1098/rstb.2009.0103 PMC2817223 20124341

[62] Hochberg B. Controlling the false discovery rate: a practical and powerful approach to multiple testing. J. R. Stat. Soc. 1995; 57:289–300.

[63] Sugiura M. The chloroplast genome. Plant Mol. Biol. 1992; 19:149–168. 1600166 10.1007/BF00015612

[64] Mullet J.E. Dynamic regulation of chloroplast transcription. Plant Physiol. 1993; 103:309–313. 8029331 10.1104/pp.103.2.309 PMC158985

[65] Quesada-Vargas T., Ruiz O.N., Daniell H. Characterization of heterologous multigene operons in transgenic chloroplasts: transcription, processing, and translation. Plant Physiol. 2005; 138:1746–1762. 15980187 10.1104/pp.105.063040 PMC1176443

[66] Bonnard G., Gobert A., Arrivé M., Pinker F., Salinas-Giegé T., Giegé P. Transfer RNA maturation in Chlamydomonas mitochondria, chloroplast and the nucleus by a single RNase P protein. Plant J. 2016; 87:270–280. 27133210 10.1111/tpj.13198

[67] Cohen E., Zafrir Z., Tuller T. A code for transcription elongation speed. RNA Biol. 2018; 15:81–94. 29165040 10.1080/15476286.2017.1384118 PMC5785986

[68] Tuller T., Veksler-Lublinsky I., Gazit N., Kupiec M., Ruppin E., Ziv-Ukelson M. Composite effects of gene determinants on the translation speed and density of ribosomes. Genome Biol. 2011; 12:R110. 22050731 10.1186/gb-2011-12-11-r110 PMC3334596

[69] Pan T., Sosnick T. RNA folding during transcription. Annu Rev Biophys Biomol Struct. 2006; 35:161–175. 16689632 10.1146/annurev.biophys.35.040405.102053

[70] Bevilacqua A., Ceriani M.C., Capaccioli S., Nicolin A. Post-transcriptional regulation of gene expression by degradation of messenger RNAs. J. Cell Physiol. 2003; 195:356–372. 12704645 10.1002/jcp.10272

[71] Scharff L.B., Ehrnthaler M., Janowski M., Childs L.H., Hasse C., Gremmels J., Ruf S., Zoschke R., Bock R. Shine-Dalgarno sequences play an essential role in the translation of plastid mRNAs in tobacco. Plant Cell. 2017; 29:3085–3101. 29133466 10.1105/tpc.17.00524 PMC5757275

[72] Zur H., Tuller T. New universal rules of eukaryotic translation initiation fidelity. PLoS Comput. Biol. 2013; 9:e1003136. 23874179 10.1371/journal.pcbi.1003136 PMC3708879

[73] Tuller T., Zur H. Multiple roles of the coding sequence 5′ end in gene expression regulation. Nucleic Acids Res. 2015; 43:13–28. 25505165 10.1093/nar/gku1313 PMC4288200

