# Supplementary material for: Prediction and large-scale analysis of primary operons in plastids reveals unique genetic features in the evolution of chloroplasts
Source: Nucleic Acids Res. 2019 Mar 4;47(7):3344–52. doi: 10.1093/nar/gkz151 (PMC6468310; doi:10.1093/nar/gkz151)
Supplement: Supplementary Data [file gkz151_supplemental_file.pdf]

Supplemental Information

Table S1.

organism	gene-pair	source	label	primer sequence	amplicon length
C. reinhardtii	psaC-petL	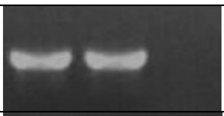	1	ACTCAATGTGTACGTGCTTGTC<br>GCACCAATAAGTAAACCTACGTAAC	885
C. reinhardtii	rpl2-rps19	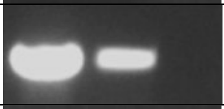	1	TCCTAACCGTAATGCTCGCA<br>AGCATGGCCACGATATGTAC	1617
C. reinhardtii	psbT-psbB	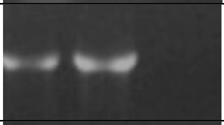	1	GGGTTTACCTTGGTATCGTG<br>CATACGTGGAGGATCTCT	2063
C. reinhardtii	rpl16-rpl14	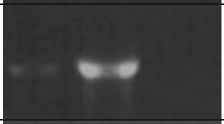	1	AGAGGAAAAGCAACACGTGG<br>TCACGACGGATACCTTTACGT	1092
C. reinhardtii	rps18-ycf3	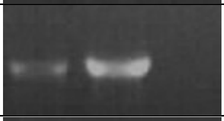	1	CGGTAACAAGCCCATTACAC<br>GACAGGACGTCTAACAGGTT	772
C. reinhardtii	trnM1-trnG1	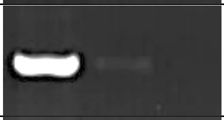	1 (weak)	GGTAGGTTCAACTCCTATACGG<br>CCGCAACATTTTCCTTGGCA	456
C. reinhardtii	trnG2-rbcL	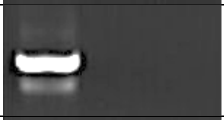	0	GAACCCACAACCTCGAACTT<br>AGACTGGTGTTCAATGCCAG	635
C. reinhardtii	petD-trnR1	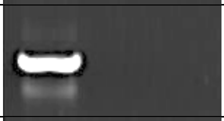	0	ATGGGTCACAACACTTACGG<br>TAGCCATGTGCTCTAGTCCA	726
C. reinhardtii	trnS2-ycf12	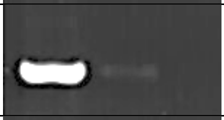	1 (weak)	AGGGATTCGAACCCTCGTAA<br>CTGCTGGTCCTCTTGTTGTT	546
C. reinhardtii	ccsA-trnL2	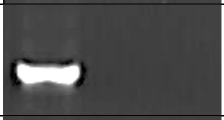	0	GTTTGCTTGAAACCCGTCAG<br>AATGGTAGACGCTACGGACT	681
C. reinhardtii	rpoC1a-trnV	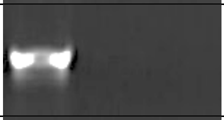	0	CCTTGTAGAGGACTGTTGCCAT<br>ACTCAGTCGGTAGAGTGATTGC	669
C. reinhardtii	tufA-trnE1	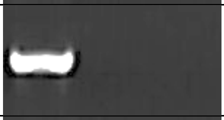	0	GCGTTTCGCTATTCGTGAAGGT<br>ATTCGAATCCGCGTTTTCTCCG	624
C. reinhardtii	trnL1-petB	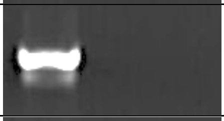	0	GCCTTTCAGCACTGGTTCCTAA<br>CAAGTTGGTTACTGGGCGGTTA	777
C. reinhardtii	chlN-psbA	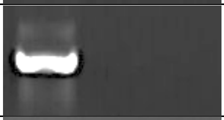	0	ACCACGTTAATGCGTCGTAG<br>TCCACTCACAAAAACGAGCC	1830
C. reinhardtii	trnS1-clpP	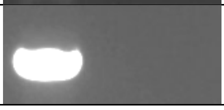	0	CGAAGCCACGCCAATTTTCA<br>AGCTGCATCTGCTGCTTCTT	1117

C. reinhardtii	psbK-tufA	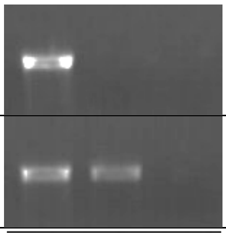	0	ACTTCCTGAAGCATACGCAC<br>CGTGGTCAACGTGACCAATA	908
C. reinhardtii	rps8-psaA		1	ACGCCTGAAGGTCTAATGACTG<br>TGCACCGTATAGGAGTTGAACC	995
C. reinhardtii	rbcL	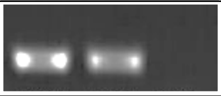	-	GCTATGCACGCGGTTATTGA<br>AACACCTGGCATTGAACACC	237
C. reinhardtii	psbJ-atpI-psaJ-rps12	(1, 2)	1	-	--
C. reinhardtii	rps12-orf1995	(1, 2)	0	-	-
C. reinhardtii	psbD-psaA	(3)	1	-	
C. reinhardtii	atpA-psbI-cemA-atpH	(2, 4)	1	-	--
C. reinhardtii	psaB-atpA	(2, 4)	0	-	-
C. reinhardtii	atpH-atpF	(2, 4)	0	-	-
C. reinhardtii	rrnS-trnI-trnA-rrn7-rrn3-rrnL-rrn5	(2, 5)	1	-	-
C. reinhardtii	rps4-rrnS	(2, 5)	0	-	-
C. reinhardtii	rrn5-psbN	(2, 5)	0	-	-
C. merolae	rpl23-rpl2	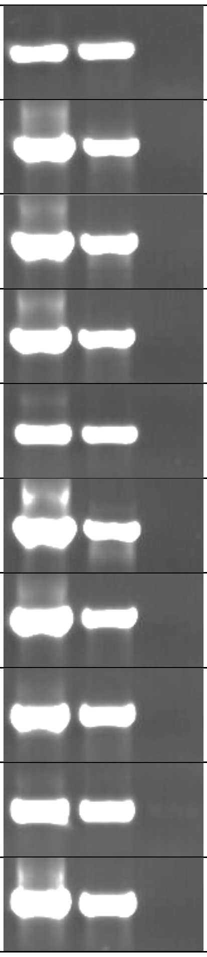	1	ATGCGCAAACCAGACTTGAG<br>TAAACGTGCACTTCGGTTCG	310
C. merolae	rpl2-rps19		1	ACGTGTCGTCATAAAGGTGG<br>ACAATGGTAGACGACCTTGC	799
C. merolae	psbD-psbC		1	TCTCCAGCAAATAGCATGGG<br>CAGGCAAAGCCAGTTGATTC	864
C. merolae	rps3-rpl16		1	ACACATCCAGTGGGATTTCG<br>CGTTCCTTTGCTTGAAGAGC	741
C. merolae	psbT-psbH		1	GAGAACCTCCAAGAATAGCG<br>CATGATTGGAGTTGTACCCC	305
C. merolae	psaA-psaB		1	GATTTCTTGTGGGCACAAGCGTCA<br>TGACCTGTCCAAGCTAAGCTGCTT	891
C. merolae	rps11-rpoA		1	CGAACCGATAGAAAGGGAAG<br>CGCAGTTACTATAGCAGGTC	717
C. merolae	atpE-atpB		1	TGAGACAGCTTGAACACGTG<br>CAACCTTTCTTCGTGGCTGA	557
C. merolae	psbF-psbE		1	ACAGTAGGGACGGCTAAACCAT<br>CAACAGGCGAACGACCTTTTTC	322
C. merolae	petB-petD		1	ATAGCAGACGACATCACGAG<br>GAGCGTCCGATAAATCTGGT	646
C. merolae	ORF45-ORF60		0	GGGTGAACGAAGAGACTTGCGAAT	194

		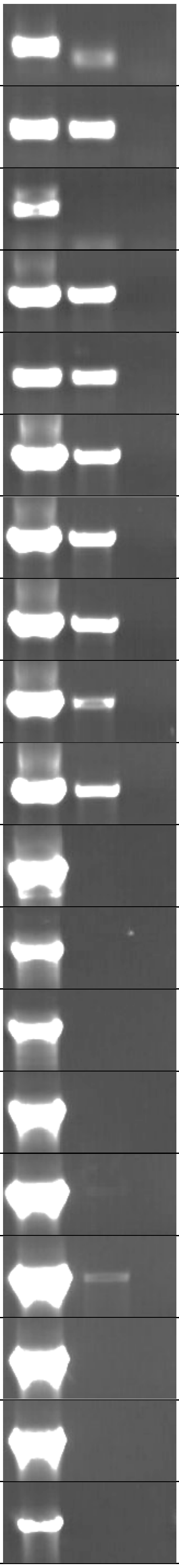		GTAGTAGTTGGGCTGGAGATACTCTC	
C. merolae	trnE-trnP		1	GGGAATTCGAATCCCCGTTA<br>GATGTCGCAGGTTCAAATCC	224
C. merolae	ORF44-secA		0	TACACCTTGTTGGAAGAGGC<br>AATACTCTTGCTGCCCCTTC	470
C. merolae	petJ-ycxr		1	ATTCAACAAGTATGGGCCGC<br>CTGCAATCCAGGCAATTTGC	583
C. merolae	ycf17-menB		1	AACCCTAACATAGCAAGGCG<br>CGTAGAAAAGCCCTTGACGA	321
C. merolae	menE-ORF138		1	AGTTACGGCACCACAGAAAC<br>CCAACCTTGGGCTTGCTTTA	868
C. merolae	psbW-rps1		1	GACAAACTGGTACGGCAGTA<br>CCACTTGCACTCTCATTCCA	883
C. merolae	ycxr-rps20		1	TAGCCTCAGCGCTAGTCTTT<br>GCGTTGAACGGCTATGAGAT	874
C. merolae	clpC-ycf17		1	ACAAAGCCAGAACCTCTTCC<br>TGGTCGCCTTGCTATGTTAG	882
C. merolae	ftsH-ycf86		1	CTGTGTTTGGAGACAGGCAA<br>GTTCTTCATCGGTACACGCT	823
C. merolae	ycf62-trnT		0	AGGCAGTATGCGCAGTACAA<br>TAATCAGCCGGTTGCAGGTT	2482
C. merolae	gltB-cpcA		0	AGGCACACAAGCCACTTTTC<br>GCTGCTGCAAGTGAAGCTAA	2497
C. merolae	ycf29-dnaB		0	GTTGAATAAGCCTCTGGCAG<br>GGACCCTGTGGACAATTCTA	2485
C. merolae	hupA-rpl3		0	TTCAAAACAATACCGCGCCG<br>TGACATTGGACCACGTGCAA	2487
C. merolae	ycf65-trnR		1 (weak)	CAACAACGGATGCCTGGAAT<br>TCGTAGCCACGTGCTCTTAT	1955
C. merolae	ycf33-psbN		1 (weak)	AACGACGACAACTCCAACAG<br>CGCGATCCATTTGAGGAACA	1877
C. merolae	cpcG-psaK		0	CCTTGCCAAGCAAATTGTGG<br>AGGTCGTAGCGATTTAACGC	2240
C. merolae	menF-menA		0	CCATGTTGATGACAGAAGCC<br>GTCATTAACCCAATTGGCCC	2313
C. merolae	menD ycf83		0	GCACTTGTATCACGGAGTTC<br>AGGTCAAACCCTACCTGAAC	2999
C. merolae	ORF60 trnL		0	TAGAGAGTATCTCCAGCCCA	2670

		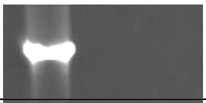		TGTGAGAGTTCGAGTCTCTC	
C. merolae	psaI psaL	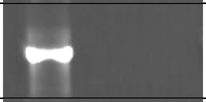	0	TATTAGTACCTACGGTGGGC<br>GGTGTAGCTAAATGGCCTAC	2372
C. merolae	ycf82 psbB	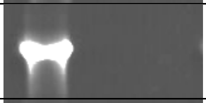	0	ACAGAAAATGGGTTGGAGGG<br>CCTTGTCGCCACATAGGATT	2860
C. merolae	preA petA	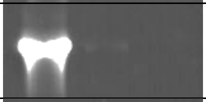	1 (weak)	ACTTGATAGGCGTCTTGCGT<br>AGCCTTTTGTGTCACCGTCT	2858
C. merolae	trnL trnR	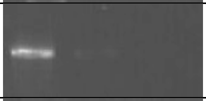	1 (weak)	ACTCGCAAAAAACATGCCCC<br>CTAAGGGATTAGGACAGGAACC	1956
C. merolae	rps4 rbcL	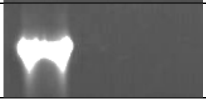	0	CGTCGTTTAGGCATCTTACC<br>GTTCTTGTACGGATTGAGCC	2277
C. merolae	rrn23-rrn5	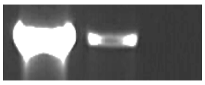	1	GTCGATGGAAAACAGGCG<br>GTCGGAGTGGATCATGAGT	1480
C. merolae	rbcL	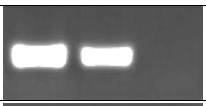	-	AAAACTTTCCAAGGGCCAGC<br>ACGATCTCTCCAACGCATGA	216
P. tricornutum	rpl23-rpl2	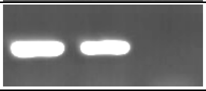	1	AATACATGCCGTTTGCCACG<br>ACCGCGATGACGAACAGTAA	307
P. tricornutum	rpl2-rps19	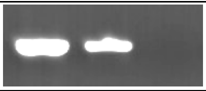	1	CCAAATCGTGGCGGACAAA<br>GTTGATGAACGAGACCAAGTTG	546
P. tricornutum	rps3-rpl16	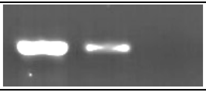	1	TTGCATTTCGTCGCGCAA<br>TAATCTACCGCCCCTTTACC	558
P. tricornutum	psbC-psbD	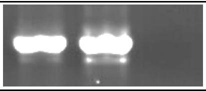	1	ATTTCCACTCCACCAAGCGA<br>GTGTTGCTGCAATCTTCCGT	597
P. tricornutum	psbH-psbT	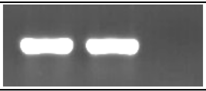	1	AATTGGAGTTGTTCCCCAACCTGG<br>GCCGTATTTTTCAGAGAAACACCTCG	370
P. tricornutum	psbF-psbL	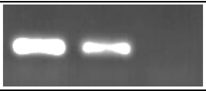	1	CCGTTGGTTAGCAATTCATGG<br>TCCCCAATAAAGAGAAGTTCTATTCA	184
P. tricornutum	rps11-rpoA	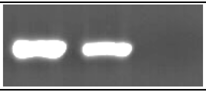	1	GGAGATACAATTTCCTGGGC<br>GCTACACCACCTAAATCACC	457
P. tricornutum	atpE-atpB	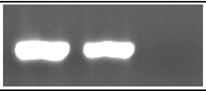	1	TTCACAGCTTCAAGACGAGC<br>GCACGTGCACGTAAAGTAGA	614
P. tricornutum	psbE-psbF	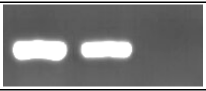	1	TACGGGTGAACGTCCGTTTT<br>TCCATGAATTGCTAACCAACGG	323
P. tricornutum	petD-petB	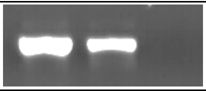	1	AAAGTTAGACTCGCAAGCGG<br>TGGCGGCAGCAGTATTAATG	539
P. tricornutum	trnH-syfB	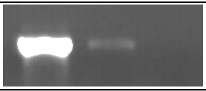	1 (weak)	AATCACAACCCGCTGCCTTA<br>TTTGGGCAACTTCACCCACT	769
P. tricornutum	trnM-rpl19	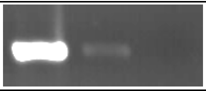	1 (weak)	CGTTTTCCTCTGGAACGTCA<br>CGCTCGACGAACTTTTGATG	428
	ycf16-atpI		1	GGACCCGAAAAGTCGATTA	656

P. tricornutum		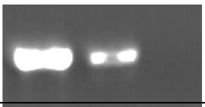		GCCCTCCGGCAATTCTATTA	
P. tricornutum	ycf90-trnE	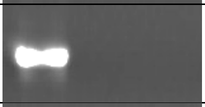	0	GCTAAAGTTAGCCCATCCTG<br>CAGACAGGGATTCGAATTCC	630
P. tricornutum	trnY-trnI	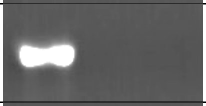	0	GTTAAAGGGGGCGGATTGTA<br>TTTGAACCTACGACCTTGGG	514
P. tricornutum	acpP-trnP	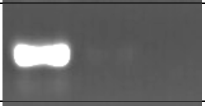	1 (weak)	TGGAAGAGAATTGGGAGCTG<br>CAGGTGCTCTACCAAACTGA	340
P. tricornutum	ycf89-trnP	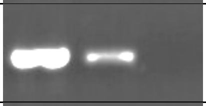	1	TTGGAGGCCAGAATGTTCTG<br>AGCTCAGTTTGGTAGAGCAC	445
P. tricornutum	trnF-rpl12	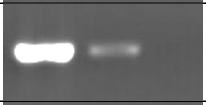	1	GCGGAGAATGGATTTGAACC<br>GCTTGATGAAGTTCCAGCTG	459
P. tricornutum	trnV-ycf33	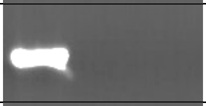	0	TGAACCTGTGACCTTCTGC<br>GGACTAATATTTCACGTTATCCTAGA	523
P. tricornutum	trnT-trnY	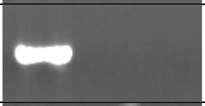	0	AGCTCAATGGTAGAGCAACG<br>GATTTACAATCCGCCCCCTT	635
P. tricornutum	rbcL	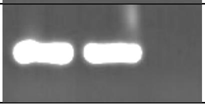	-	TCACAACCCTTCATGCGTTG<br>CCAAATCGCAGCTGATTGGA	225

Operon data of algal plastomes. Each row represents the transcription-state of a specific gene-pair / gene-cluster. Data retrieved from the RT-PCR is supplemented with a gel-profile of the three controls (DNA:cDNA:RNA, from left to right), the primers used, and the amplicon length. Known operons are supplemented with the relevant references. OPs are denoted as '1', whereas NOPs are denoted as '0'.

Table S2.

A. CDS group.

<table>
<tr><th>Feature ranking</th><th>boxplot</th><th>explanation</th></tr>
<tr><td>**1**</td><td>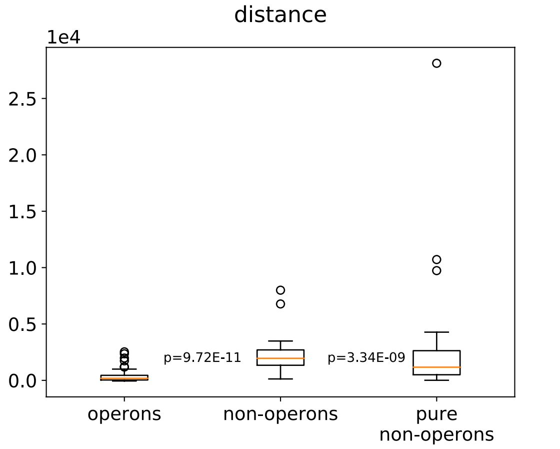
</td><td>The intergenic spacer between OPs is shorter compared to NOPs</td></tr>
<tr><td>**2**</td><td>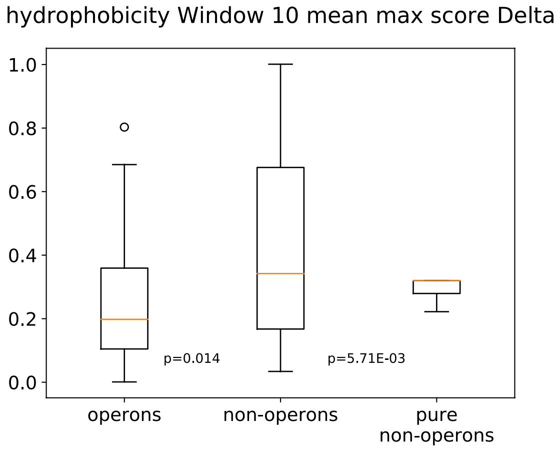
</td><td>The most hydrophilic area of two adjacent genes is more similar in OPs compared to NOPs</td></tr>
<tr><td>**3**</td><td>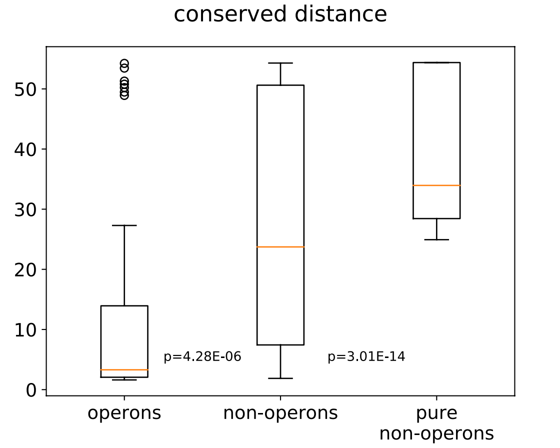
</td><td>OP genes are found in closer proximity (the number of genes between them) in other plastomes compared to NOP genes</td></tr>
</table>

4

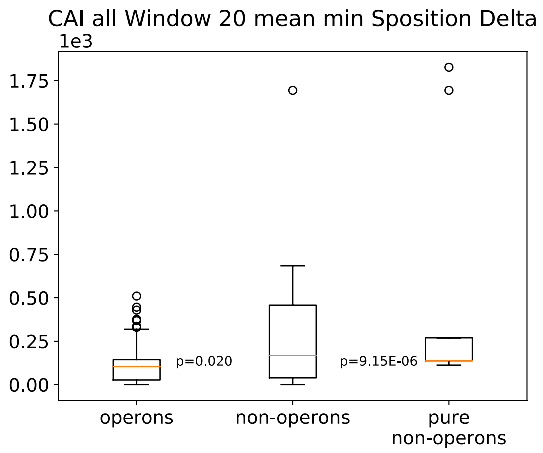

The position (from START) of the rarest codon usage window is more similar in OPs compared to NOPs

5

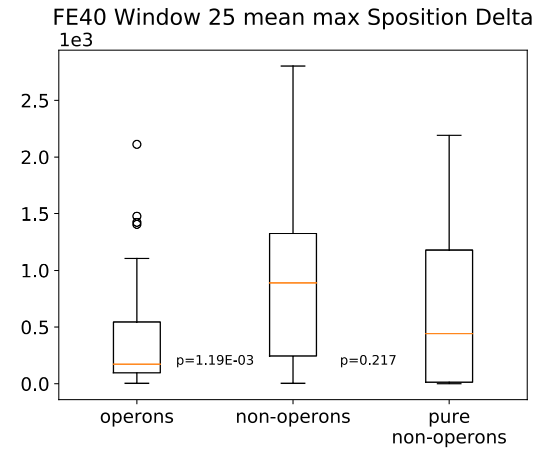

The position (from START) of the most lose RNA structure is more similar in OPs compared to NOPs

B. Mixed group.

Feature ranking	boxplot	explanation
**1**	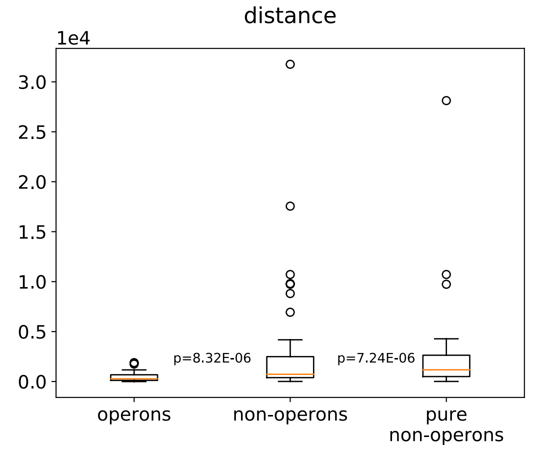	The intergenic spacer between OPs is shorter compared to NOPs

2

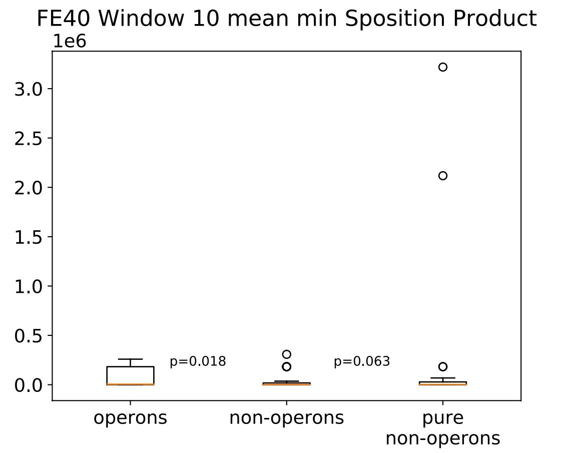

The position (from START) of the tightest RNA structure is more distant in OPs compared to NOPs

3

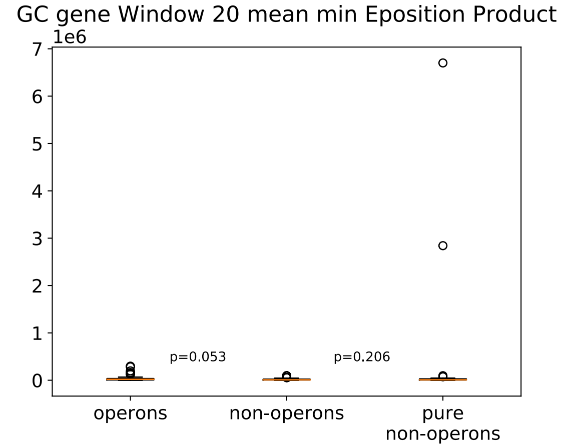

The position (from END) of the lowest GC content is more distant in OPs compared to NOPs

4

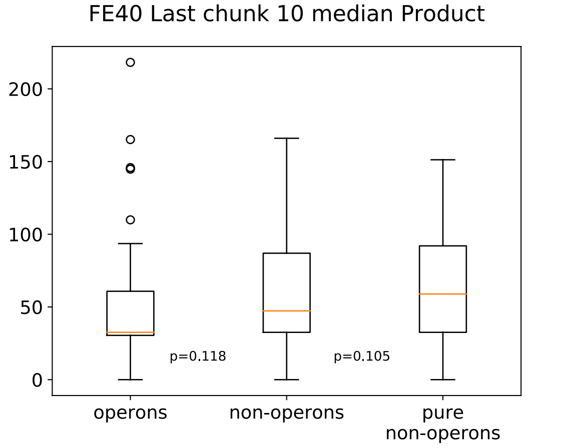

The RNA structure near the end of the gene is tighter in OPs compared to NOPs

5

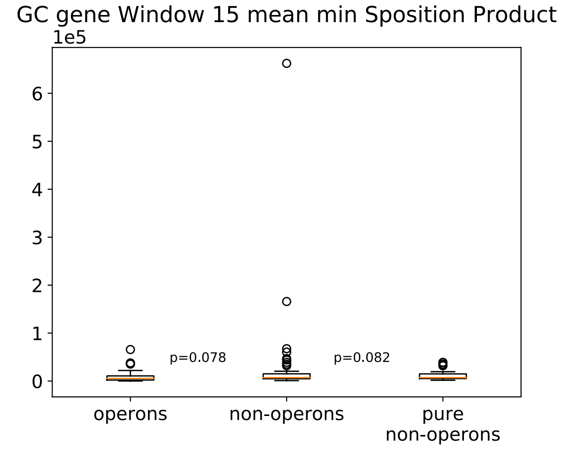

The position (from START) of the lowest GC content is closer in OPs compared to NOPs

**6**	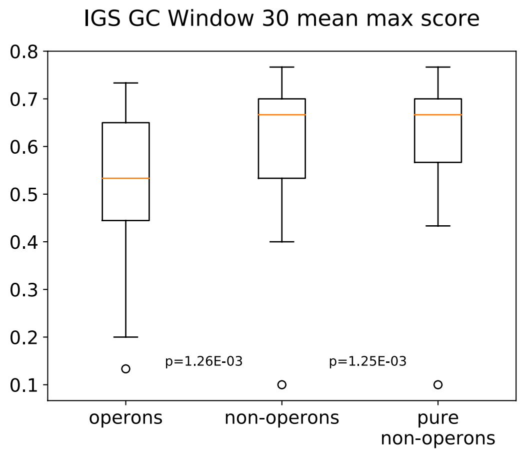	The GC content of the IGS between OPs is lower compared NOPs

Features selected to appear in the final model. The feature ranking column indicates the feature's importance ranking, as derived from the scikit-learn random-forest classifier for the final features included in the model. Each of the boxplots corresponds to a selected feature. **(A)** CDS gene-pairs. **(B)** Mixed gene-pairs (combination of tRNAs, rRNAs and CDSs). The 'operons' and 'non-operons' groups refer to empirical gene-pairs that were collected by the RT-PCR and published data; 'pure non-operons' refers to predicted non-operonic gene-pairs that are comprised of adjacent genes that are not found in any upstream or downstream polycistronic units (*i.e.* both genes are monocistrons). As our empirical operon data covers only a part of the plastomes' gene pairs, and is not necessarily continuous, the 'pure non-operons' could only be achieved by combining the empirical data with predictions. P-values between 'operons' to 'non-operons', and 'operons' to 'pure non-operons' are found between each group, respectively, and were calculated using a two-tailed Wilcoxon rank-sum test.

Table S3.

test	gene-type	accuracy	true positive rate	true negative rate
**cross-validation**	**CDS group**	0.88 ± 0.01 (N=121)	0.9 ± 0.01 (N=94)	0.8 ± 0.02 (N=27)
	**mixed group**	0.79 ± 0.02 (N=92)	0.82 ± 0.02 (N=43)	0.78 ± 0.02 (N=49)
	**overall (weighted)**	0.84 ± 0.01 (N=213)	0.87 ± 0.01 (N=137)	0.79 ± 0.02 (N=76)
**test on *C. merolae***	**CDS group**	0.83 ± 0.08 (N=30)	0.83 ± 0.04 (N=19)	0.82 ± 0.15 (N=11)
	**mixed group**	0.76 ± 0.1 (N=6)	1 (N=4)	0.3 ± 0.3 (N=2)
	**overall (weighted)**	0.81 ± 0.08 (N=36)	0.85 ± 0.03 (N=23)	0.74 ± 0.17 (N=13)

Classifiers' metrics. Each round of training and cross-validating/testing yielded an accuracy score (*i.e.* the ratio of correct predictions on the cross-validation/test group), a TPR (*i.e.* sensitivity/recall/coverage - the ratio of correct predictions on OPs) and a TNR (*i.e.* specificity/precision - the ratio of correct predictions on NOPs). All values represent the mean ± STD over ten bootstrap rounds of training and cross-validating/testing. The overall values represent the weighted mean values of each classifier. 'N' represents the number of gene-pairs sampled.

Figure S1.

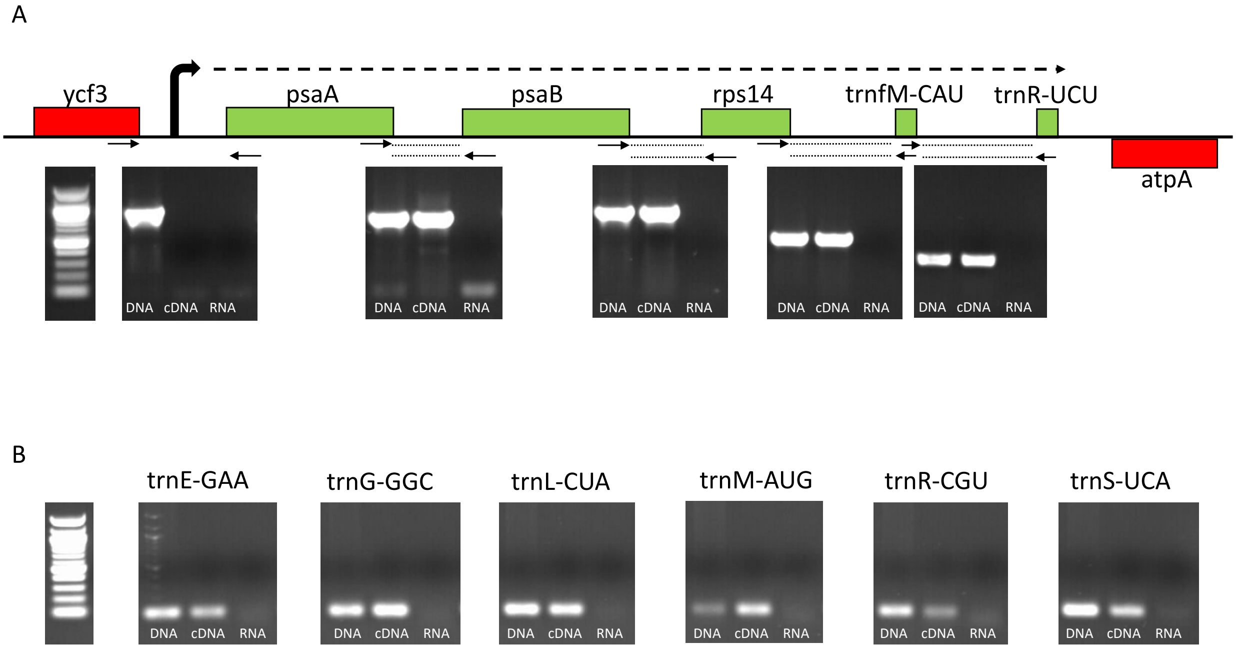

Reliability analysis of the RT-PCR method. **(A)** Identification of the known psaA operon derived from the *H. vulgare* plastome by RT-PCR. Genes colored in green are co-transcribed and are part of the psaA polycistron, whereas red genes are adjacent to the psaA operon and are not part of it. The primary transcript of the psaA operon as described in (6) is shown as a dashed line. **(B)** RT-PCR amplification of the full cDNA transcript of six tRNA genes derived from the *C. reinhardtii* plastome. See supplemental methods (Primer design and RT-PCR) for information on the primers used in these analyses.

Figure S2.

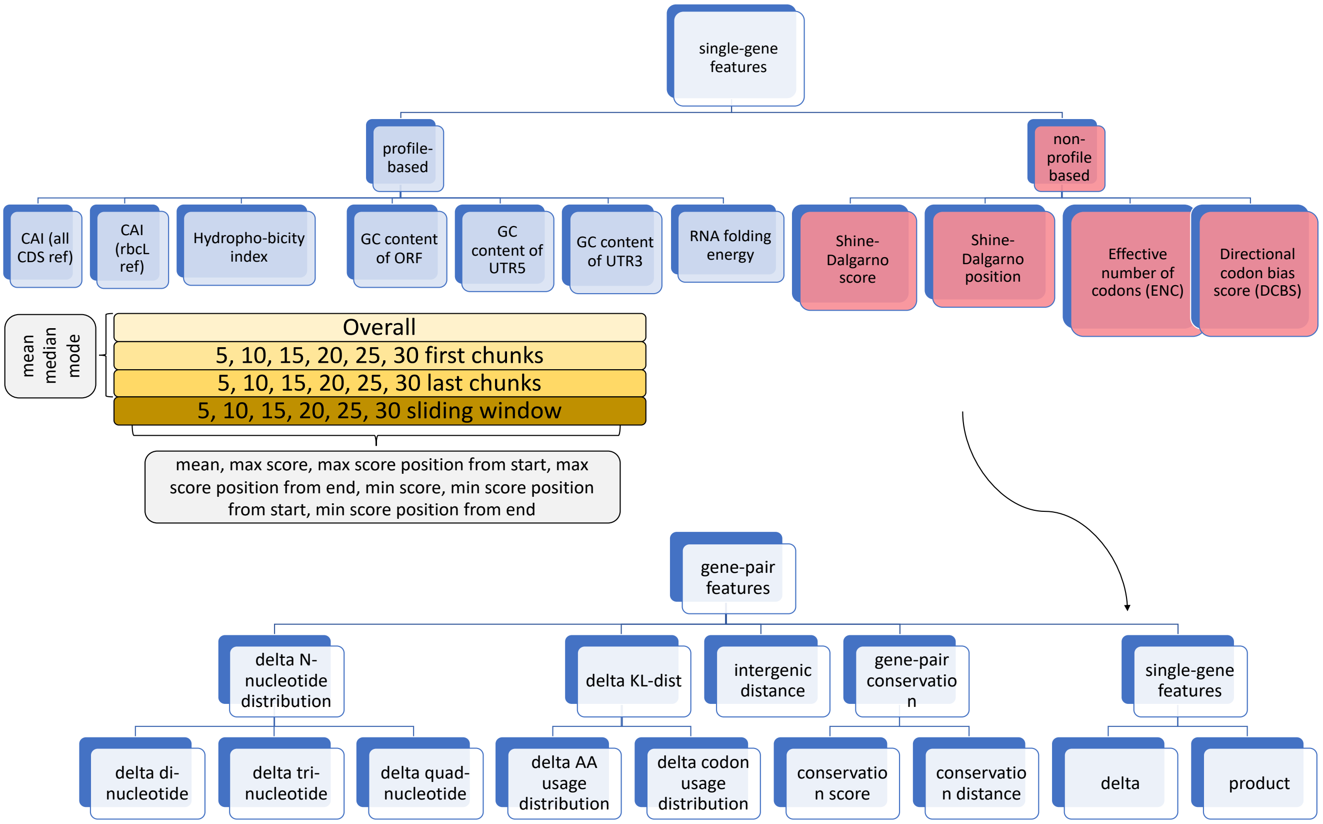

Feature engineering. Our model was initiated with roughly 1,100 features, schematically described in summary in this figure. Most of the features were calculated for single genes separately (*e.g.* GC%, mean hydrophobicity etc.) and then the margin and product of two adjacent genes were taken as final features. However, some features were initially computed on gene pairs (*e.g.* intergenic distance). All features were calculated based on sequence features alone, and were ultimately fed to the feature selection algorithm.

Figure S3.

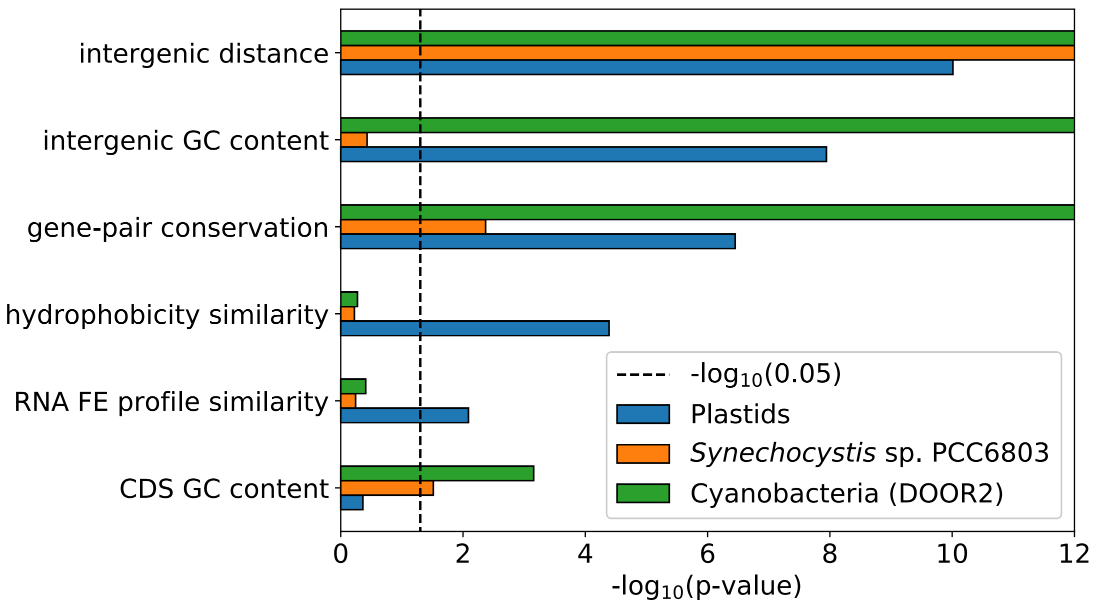

Selected operon characteristics in plastids and cyanobacteria. The horizontal bars depict how well a specific feature separated OPs from NOPs in either real labels of plastids (blue; *C. reinhardtii*, *C. merolae*, *P. tricornutum* and *H. vulgare*), real labels of *Synechocystis* sp. PCC6803 (orange) and DOOR2 predicted labels of six cyanobacteria species (green; *Nostoc azollae*, *Acaryochloris marina*, *Cyanothece* sp. ATCC, *Trichodesmium erythraeum*, *Gloeobacter violaceus* PCC 7421 and *Synechococcus elongatus* PCC 6301). P-values were calculated using a two-tailed Wilcoxon rank-sum test, and the common significance threshold is given. The directionality of the significance (whether OP values are larger/smaller than NOP values) is: (i) intergenic distance – smaller in operons, (ii) gene-pair conservation – larger in operons, (iii) RNA FE profile similarity – larger in operons, (iv) intergenic GC content – smaller in operons, (v) CDS GC content – smaller in operons, and (vi) hydrophobicity similarity – larger in operons.

Figure S4.

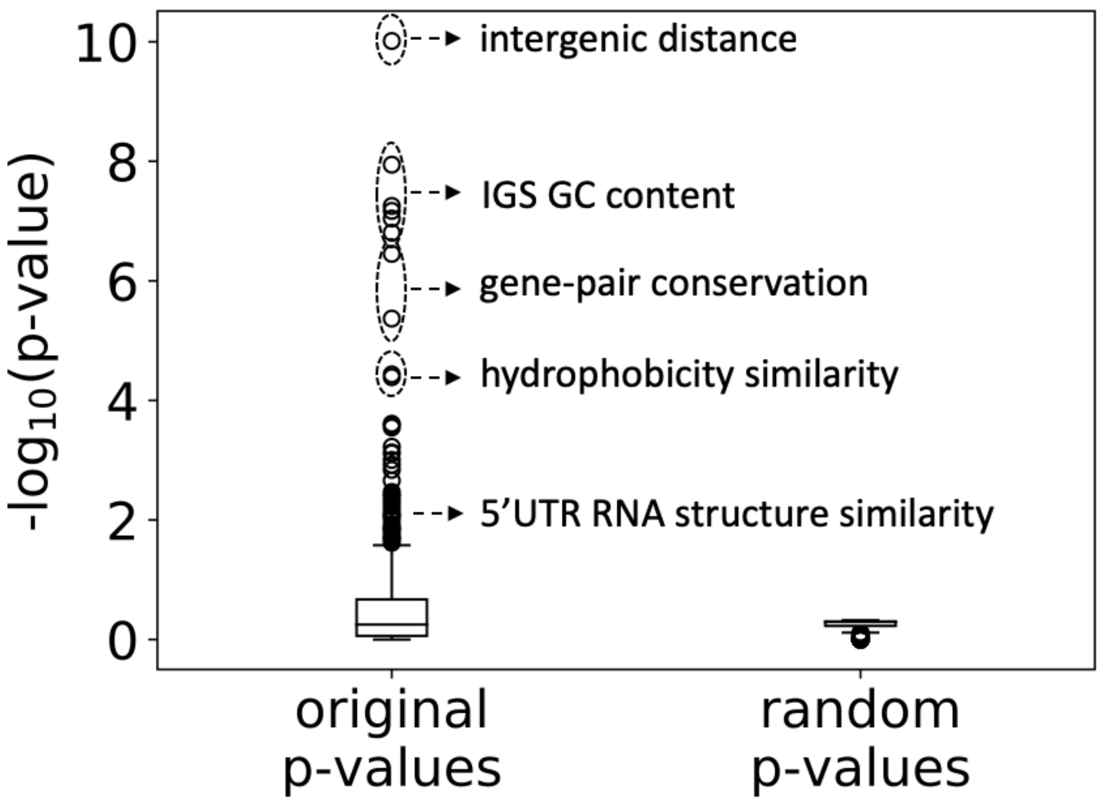

P-values testing the separation (between OP and NOP) efficiency of each feature in the original data-set (left boxplot) and random data-set (right boxplot). The random data-set was created by permutating the feature labels (OP/NOP) 1,000 times and averaging the results for each feature. P-values are given as $-\log_{10}$(p-value), and were calculated using a two-tailed Wilcoxon rank-sum test, which facilitates the computation of low values for a large data-set (in comparison to the permutation test used in Figure 2). Dashed circles that contain multiple points represent a same-type feature cluster.

Figure S5.

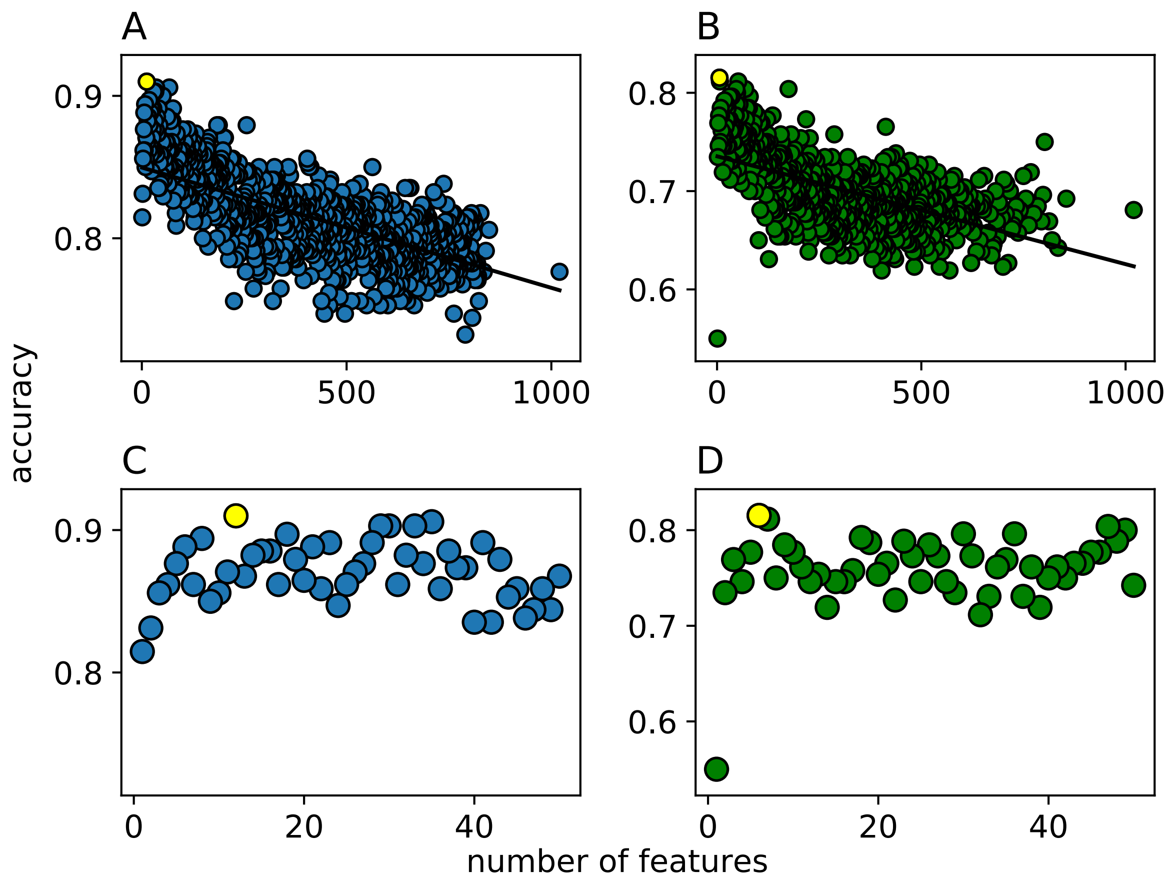

Wrapped backward elimination feature selection. The number of features used in the model against the total model accuracy in the CDS group **(A)** and the mixed group **(B). (C,D)** Close-ups of the final iterations of (A) and (B), respectively. The yellow dots signify the initial selected feature-set, from which semi-redundant features were removed in order to formulate the final feature composition of the predictor.

Figure S6.

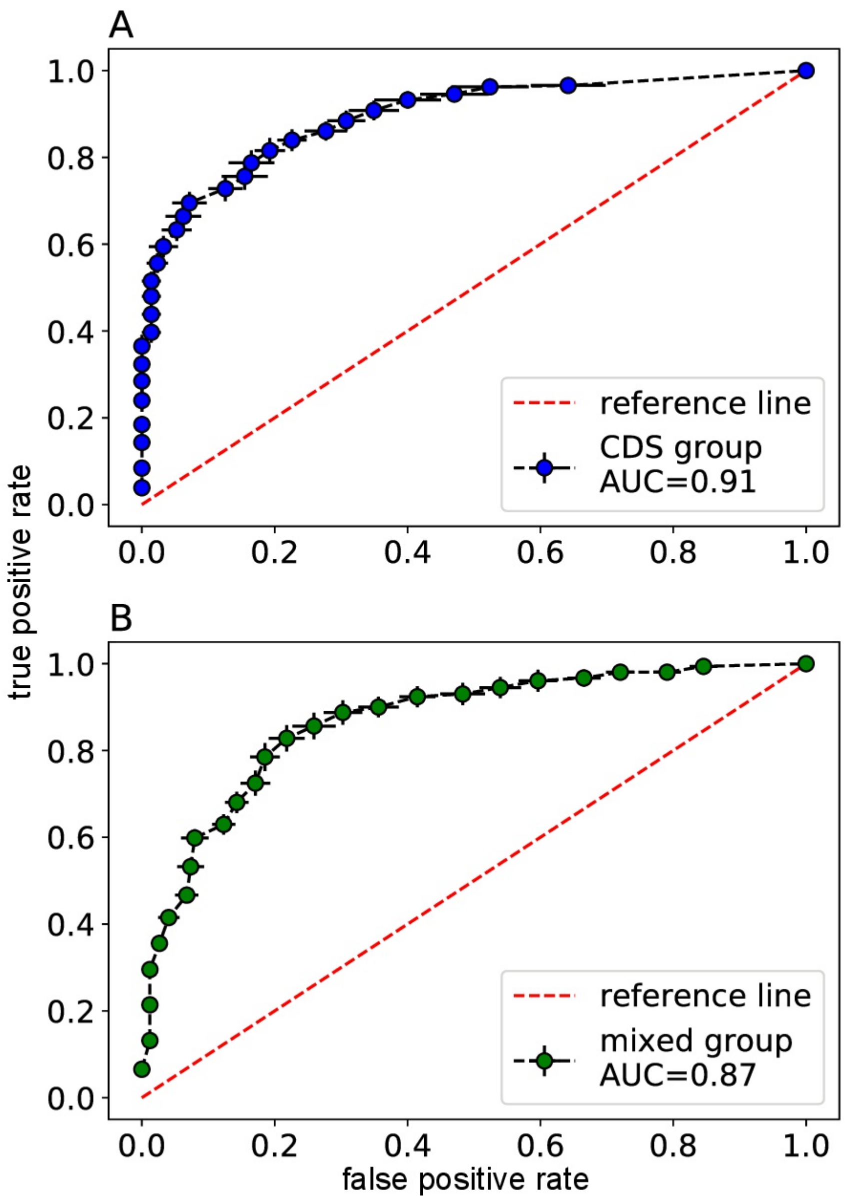

Receiver operating characteristic (ROC) curves. Thresholds, true positive rates (TPR) and false positive rates (FPR) were calculated for each of the 20 classifiers - (A) CDS group and (B) mixed group - using sklearn's 'roc_curve' function (7, 8). The area under the curve (AUC) values were calculated using sklearn's 'roc_auc_score' function (7, 8). The dashed red reference lines represent hypothetical random classifiers. Each dot contains a threshold-specific Y-standard-error and a X-standard-error for TPR and FPR variation between classifiers, respectively.

Supplemental methods

Cultures and growth conditions:

All species were grown under standard conditions:

- *C. reinhardtii* CC124 wild-type strain cultures were grown in Tris-Acetate-Phosphate (TAP) medium at 25°C under continuous cool daylight and cool white fluorescent lights (90 µE m-2 s-1) stirring in 100 mL Erlenmeyers capped with silicone sponge enclosures.
- *C. merolae* 10D wild-type strain cultures were grown in Modified Allen's medium 2 (MA-2, pH 2.8) under 42°C continuous daylight and cool white fluorescent lights (50 µE m-2 s-1) stirring in 100 mL Erlenmeyers capped with silicone sponge enclosures.
- *P. tricornutum* wild-type strain cultures were grown in Artificial Sea Water (ASW) 0.5M medium at 25°C under continuous daylight and cool white fluorescent lights (90 µE m-2 s-1) stirring in 100 mL Erlenmeyers capped with silicone sponge enclosures.
- *H. vulgare* line 'Golden Promise' was grown for 11 days in soil at 22°C in a growth chamber with a photoperiod of 16h (150 µE m-2 s-1).

DNA extraction, RNA extraction and cDNA synthesis:

DNA from all algal cultures was extracted as described in (9). For *H.* vulgare, 50 mg of fresh leaves were taken for total DNA extraction using the OMEGA E.Z.N.A SP Plant DNA kit DNA. 100 mg of the cell pellet was taken for total RNA extraction using RNeasy plant Mini Kit (QIAGEN 74903). The cell suspension was lysed in a Minilys tissue lyser for 90 seconds (Bertin technologies). Total RNA was treated with Turbo DNA-free™ Kit (Ambion AM1907) according to the manufacturer's recommendation, followed by PCR and gel electrophoresis quality check. The RNA concentration was determined using a NanoDrop® ND-1000 Spectrophotometer. 1 µg of purified RNA from each sample was used for complementary DNA (cDNA) synthesis using High capacity cDNA Reverse Transcription Kit (ABI 4368814) that was performed with random primers according to the manufacturer protocol.

Primer design and Reverse-Transcription PCR (RT-PCR):

To retrieve data on plastid operons for *C. merolae*, *P. tricornutum* and *C. reinhardtii*, each plastome (NC_004799, NC_008588 and NC_005353, respectively) was organized as a list of adjacent gene-pairs. From each organism 20 to 40 gene-pairs were selected for RT-PCR analysis. Specific primers were designed for each chosen gene-pair; the forward annealed to the 5' gene whereas the reverse primer annealed to the 3' gene (see Table S1). Primers for assessing the reliability of the RT-PCR method (Figure S1) are shown below:

organism	gene-pair / gene	Primer sequence	amplicon length
H. vulgare	ycf3-psaA	CCGAGGAGAACAGGCCATTCTA<br>GTGTTCTTGAGAAATGGCCGGG	936
H. vulgare	psaA-psaB	ATGCAGTCGGATGTTTGGGGTA<br>AGCAGCACCTCCTCGAGTAAAG	774
H. vulgare	psaB-rps14	TTATGGCAGGGCAACGTTTCAC<br>CCAACTGGATCTTGTTGCACCC	859
H. vulgare	rps14-trnF	AGGGAGAAGAAGCGGCAGAAAT<br>GAATCGAACCCGCAACCCCAAG	508
H. vulgare	trnF-trnR	AGAGCAGTTTGGTAGCTC<br>CGTCCAATAGGATTTGAACC	294
C. reinhardtii	trnE-GAA	GCCCCCATCGTCTAGAGG<br>TACCCCCAGCGGAATTCGAA	73
C. reinhardtii	trnG-GGC	GCGGACATAGCTCAATGGTA<br>AGCGGACAACGGGAGTCGAA	72
C. reinhardtii	trnL-CUA	GGGGATATGGCGGAATGGTA<br>TGGGGAAAGAGGGACTTGAA	85
C. reinhardtii	trnM-AUG	GCACTGTTGGCCGAGCGGAT<br>TGCACCGTATAGGAGTTG	73
C. reinhardtii	trnR-CGU	GAGCTTGTAGCTCAGTGG<br>CGAGCCAGGAGGGTTTCGAA	74
C. reinhardtii	trnS-UCA	GGAAAGGTGGCAGAGTGGTT<br>CGGAAAGGGAGGGATTCGAA	87

Gene-pair conservation

To compute gene pair conservation, we first arranged a database of homologs. This was achieved by applying an iterative algorithm to our whole database. In the case of chloroplasts,

this database included all the plastomes downloaded (see list of names and NCBI IDs in https://www.energylabtau.com/cppod). In case of cyanobacteria, this database included 54 cyanobacteria species:

NC_009925	Acaryochloris marina MBIC11017
NC_019771	Anabaena cylindrica PCC
NZ_AP018174	Anabaenopsis circularis NIES-21
NZ_CM001632	Arthrospira platensis C1
NZ_AP018307	Aulosira laxa NIES-50
NZ_AP018207	Calothrix brevissima NIES-22
NC_019697	Chamaesiphon minutus PCC
NZ_AP018281	Chondrocystis sp. NIES-4102
NC_019695	Chroococcidiopsis thermalis PCC
NC_019753	Crinalium epipsammum PCC
NC_019776	Cyanobacterium aponinum PCC
NC_019675	Cyanobium gracile PCC
NC_010546	Cyanothece sp. ATCC
NZ_LYXA01000001	Cylindrospermopsis raciborskii CS-505
NC_019757	Cylindrospermum stagnale PCC
NC_019780	Dactylococcopsis salina PCC
NZ_AP018316	Dolichospermum compactum NIES-806
NZ_KB904821	Filamentous cyanobacterium ESFC-1
NZ_AP017305	Fischerella sp. NIES-3754
NZ_KB235930	Fortiea contorta PCC
NZ_AP018233	Fremyella diplosiphon NIES-3275
NZ_KB235958	Geitlerinema sp. PCC
NZ_CM001775	Geminocystis herdmanii PCC
NC_022600	Gloeobacter kilaueensis JS1,
NC_019745	Gloeocapsa sp. PCC
NZ_CP017675	Gloeomargarita lithophora Alchichica-D10
NZ_CP021983	Halomicronema hongdechloris C2206
NC_019779	Halothece sp. PCC
NZ_AP014642	Leptolyngbya boryana dg5
NZ_JTHE01000274	Lyngbya confervoides BDU141951
NZ_JH992901	Mastigocladopsis repens PCC
NC_019738	Microcoleus sp. PCC
NZ_CP012375	Microcystis aeruginosa NIES-2481,
NZ_MKZS01000001	Moorea bouillonii PNG
NZ_JH976537	Nodosilinea nodulosa PCC
NZ_BDUB01000001	Nodularia sp. NIES-3585,
NZ_AP018180	Nostoc carneum NIES-2107
NZ_CP019636	Nostocales cyanobacterium HT-58-2,
NC_019693	Oscillatoria acuminata PCC
NZ_CM001633	Oscillatoriales cyanobacterium JSC-12
NZ_CM002803	Planktothrix agardhii NIVA-CYA
NZ_KB235922	Pleurocapsa sp. PCC
NZ_KB235914	Pseudanabaena sp. PCC
NZ_AP018317	Raphidiopsis curvata NIES-932
NC_019678	Rivularia sp. PCC
NZ_KQ976354	Scytonema hofmannii PCC
NZ_AP018314	Sphaerospermopsis kisseleviana NIES-73
NZ_KV878783	Spirulina major PCC
NC_019748	Stanieria cyanosphaera PCC
NZ_AUMZ01000001	Synechococcus sp. 60AY4M2
NZ_CP007542	Synechocystis sp. PCC
NZ_AP018248	Tolypothrix tenuis PCC
NC_008312	Trichodesmium erythraeum IMS101,

Description of the homolog determination algorithm:

- Initiation: a single gene was given a unique name and defined as the database
- Iteration: one gene was compared to the database, using BLAST (https://www.ncbi.nlm.nih.gov/books/NBK279690). If a homolog (threshold: E-value < E-5) was found, the new gene was given the same name as its homolog, otherwise it was given a new unique name. At the end of this stage the new gene was appended to the database.
- Termination: Simply after all the genes were given names.

After creating this homolog list, searching for gene pair conservation is straightforward: for a given pair A-B, we iterated over all organisms in the database and looked for the genes A and B. We computed several conservation indices:

- A simple count of how many times the pair A-B was found in the database.
- The average distance of gene A from gene B, across all organisms (taking into consideration the circular structure of the genome, excluding cases where the genome was defined as linear). If both genes were missing from a certain genome – the score given was the longest distance possible for that genome (*e.g.* for a circular genome of length L, it would be L/2). If only one gene was missing from a certain genome – the score given was half of the longest distance possible for that genome (*e.g.* for a circular genome of length L, it would be L/4).
- Each of the indices measured above was also normalized; to this end we divided the result by the total number of species from the organism's group (the logic was to reduce high scores from groups with many representatives in the database). The group was determined by the given organism's taxonomy. Thus, for each organism we computed four conservation indices.

Supplemental references

1. Rymarquis,L.A., Higgs,D.C. and Stern,D.B. (2006) Nuclear suppressors define three factors that participate in both 5′ and 3′ end processing of mRNAs in Chlamydomonas chloroplasts. *Plant J.*, **46**, 448–461.
2. Jalal,A., Schwarz,C., Schmitz-Linneweber,C., Vallon,O., Nickelsen,J. and Bohne,A.V. (2015) A small multifunctional pentatricopeptide repeat protein in the chloroplast of chlamydomonas reinhardtii. *Mol. Plant*, **8**, 412–426.
3. Cavaiuolo,M., Kuras,R., Wollman,F.A., Choquet,Y. and Vallon,O. (2017) Small RNA profiling in chlamydomonas: Insights into chloroplast RNA metabolism. *Nucleic Acids Res.*, **45**, 10783–10799.
4. Drapier,D., Suzuki,H., Levy,H., Rimbault,B., Kindle,K.L., Stern,D.B. and Wollman,F. a (1998) The chloroplast atpA gene cluster in Chlamydomonas reinhardtii. Functional analysis of a polycistronic transcription unit. *Plant Physiol.*, **117**, 629–641.
5. Holloway,S.P. and Herrin,D.L. (1998) Processing of a composite large subunit rRNA. Studies with chlamydomonas mutants deficient in maturation of the 23s-like rrna. *Plant Cell*, **10**, 1193–206.
6. Zhelyazkova,P., Sharma,C.M., Forstner,K.U., Liere,K., Vogel,J. and Borner,T. (2012) The Primary Transcriptome of Barley Chloroplasts: Numerous Noncoding RNAs and the Dominating Role of the Plastid-Encoded RNA Polymerase. *Plant Cell*, **24**, 123–136.
7. Pedregosa,F., Varoquaux,G., Gramfort,A., Michel,V., Thirion,B., Grisel,O., Blondel,M., Prettenhofer,P., Weiss,R., Dubourg,V., *et al.* (2012) Scikit-learn: Machine Learning in Python. *J. Mach. Learn. Res.*, **12**, 2825–2830.
8. Liaw,A. and Wiener,M. (2014) Classification and Regression by randomForest.
9. Cao,M., Fu,Y., Guo,Y. and Pan,J. (2009) Chlamydomonas (Chlorophyceae) colony PCR. 10.1007/s00709-009-0036-9.
